# The role of interferon regulatory factor 8 for retinal tissue homeostasis and development of choroidal neovascularisation

**DOI:** 10.1186/s12974-021-02230-y

**Published:** 2021-09-20

**Authors:** Peipei Zhang, Anja Schlecht, Julian Wolf, Stefaniya Boneva, Yannik Laich, Jana Koch, Franziska Ludwig, Myriam Boeck, Adrian Thien, Carmen Härdtner, Katrin Kierdorf, Hansjürgen Agostini, Günther Schlunck, Marco Prinz, Ingo Hilgendorf, Peter Wieghofer, Clemens Lange

**Affiliations:** 1 Medical Faculty, Eye Center, University Hospital, University of Freiburg, Freiburg im Breisgau, Germany; 2 Institute of Anatomy, Wuerzburg University, Wuerzburg, Germany; 3 Cardiology and Angiology, University Heart Center, University of Freiburg, Freiburg im Breisgau, Germany; 4 Medical Center and Faculty of Medicine, Institute for Experimental Cardiovascular Medicine, University Heart Center Freiburg Bad Krozingen, University of Freiburg, Freiburg, Germany; 5 Medical Faculty, Institute of Neuropathology, University Hospital, University of Freiburg, Freiburg im Breisgau, Germany; 6 CIBSS-Centre for Integrative Biological Signalling Studies, University of Freiburg, Freiburg, Germany; 7 Medical Faculty, Center for Basics in NeuroModulation (NeuroModulBasics), University of Freiburg, Freiburg im Breisgau, Germany; 8 Signalling Research Centres BIOSS and CIBSS, University of Freiburg, Freiburg im Breisgau, Germany; 9 Institute of Anatomy, Leipzig University, Leipzig, Germany

**Keywords:** Irf8, Interferon regulatory factor 8, Retinal microglia, Choroidal neovascularisation, RNA sequencing

## Abstract

**Background:**

Microglia cells represent the resident innate immune cells of the retina and are important for retinal development and tissue homeostasis. However, dysfunctional microglia can have a negative impact on the structural and functional integrity of the retina under native and pathological conditions.

**Methods:**

In this study, we examined interferon-regulatory factor 8 (*Irf8*)–deficient mice to determine the transcriptional profile, morphology, and temporospatial distribution of microglia lacking *Irf8* and to explore the effects on retinal development, tissue homeostasis, and formation of choroidal neovascularisation (CNV).

**Results:**

Our study shows that *Irf8*-deficient MG exhibit a considerable loss of microglial signature genes accompanied by a severely altered MG morphology. An in-depth characterisation by fundus photography, fluorescein angiography, optical coherence tomography and electroretinography revealed no major retinal abnormalities during steady state. However, in the laser-induced CNV model, *Irf8*-deficient microglia showed an increased activity of biological processes critical for inflammation and cell adhesion and a reduced MG cell density near the lesions, which was associated with significantly increased CNV lesion size.

**Conclusions:**

Our results suggest that loss of *Irf8* in microglia has negligible effects on retinal homeostasis in the steady state. However, under pathological conditions, *Irf8* is crucial for the transformation of resident microglia into a reactive phenotype and thus for the suppression of retinal inflammation and CNV formation.

**Supplementary Information:**

The online version contains supplementary material available at 10.1186/s12974-021-02230-y.

## Background

Retinal microglia cells (rMG) constitute the resident myeloid cell population in the neuroretina and are critical for retinal development, tissue homeostasis and response to cell damage. During development, rMG are in intimate contact to endothelial tip cells and contribute to postnatal vascular development [16] as well as neuronal survival by modulating programmed cell death and trophic influences [56]. During adulthood, rMG interact closely with synapses to maintain synaptic structure and electroretinal function and continuously scan the local environment for danger signals associated with injury or pathogens [56]. In response to tissue damage or infection, rMG rapidly attain an activated phenotype, migrate towards the site of injury and contribute to phagocytosis, inflammation and pathological events [4, 52, 59]. As such, activated microglia cells have been found in the subretinal space of patients with age-related macular degeneration (AMD) and in particular at sites of choroidal neovascularisation (CNV) in neovascular AMD [11, 20] which is a common cause of irreversible blindness in the elderly [10, 60]. Studies on the role of rMG in the development of CNV, however, revealed conflicting results, and both detrimental and protective roles of MG for the progression of CNV have been discussed in the past [2, 14, 36, 49, 51].

The interferon regulatory factor (IRF) family of transcription factors consists of nine members that are involved in hematopoietic differentiation, oncogenesis, Toll-like and purinergic receptor signalling and expression of interferons and interferon-inducible genes [39, 57]. In particular, *Irf8* plays a pivotal role in the regulation of lineage commitment and MG cell maturation during brain development [27, 47]. Besides its essential role during development, *Irf8* is crucial for the function of resident myeloid cells in the adult steady state. As such, the deletion of *Irf8* in mice leads to a disturbed homeostasis of resident tissue macrophages in the liver, the kidney and brain including microglia and other CNS-associated macrophages [19, 25, 48, 58]. The role of *Irf8* in regulating rMG gene expression and its influence on retinal development and neuroretinal function, however, are currently unknown.

The aim of this study was to determine the function of IRF8 in retinal microglia in the healthy as well as perturbed retina. Specifically, we aimed to investigate whether IRF8 is involved in microglial cell homeostasis, neuroretinal function and pathological CNV formation. The latter is of particular interest, as PU.1 and CSF1R signalling acting upstream and downstream of IRF8, respectively, are critical for postnatal angiogenesis and formation of pathological neovascularisation in the eye [16, 54]. For this purpose, we analysed *Irf8* reporter and knockout mice by *in vivo* imaging, functional studies, flow cytometry, immunohistochemistry and RNA sequencing (RNA-seq). We found that *Irf8*-deficient MG exhibited functionally relevant alterations in gene expression patterns that were associated with a significant disruption of microglial development, normal postnatal retinal vascular and functional development, and increased CNV lesion size in the adult situation.

## Methods

### Mice

All animal experiments were authorized by the local animal care and use committee under the respective EU, national, federal and institutional regulations for animal experiments (ethical protocol numbers G14/89, G20/13). Mice were bred on a C57BL/6J background and devoid of the Crb1 mutation. *Cx3cr1*^GFP/GFP^ mice were crossed with C57BL/6J mice to generate *Cx3cr1*^GFP/+^ (*Irf8* WT) mice. *Irf8*^-/-^ mice were crossed with *Irf8*^-/-^*Cx3cr1*^GFP/GFP^ mice to obtain *Cx3cr1*^GFP/+^:*Irf8*^-/-^ (*Irf8* KO) mice [24, 26]. Phage artificial chromosome-transgenic *Irf8-*VENUS reporter mice were used to trace the expression of IRF8 [53]. *CAG::mRFP1* mice were purchased from the Jackson Laboratory (Bar Harbor, ME).

### Genotyping

Transgenic mice were genotyped according to the primers and programs shown in supplemental table 1.

### Laser-induced choroidal neovascularisation (CNV)

The laser-induced CNV model was used as previously described [15, 31, 50]. In brief, mice were anaesthetized by intraperitoneal administration of ketamine hydrochloride (100 mg/kg, Pharmacia & Upjohn, Erlangen, Germany) and xylazine (6 mg/kg, Bayer Vital GmbH, Leverkusen, Germany). Pupillary dilatation was achieved by applying 0.5% tropicamide (Bausch + Lomb, Berlin, Germany) and 5% phenylephrine hydrochloride (URSAPHARM Arzneimittel GmbH, Saarbrücken, Germany). After covering the cornea with a coverslip coated with dexpanthenol eye gel (50 mg/g, Bausch + Lomb, Berlin, Germany), three to six laser spots (488 nm, 150 mW, 100 μm and 100 ms) were applied to each eye using the VISULAS 532s Laser System (Carl Zeiss, Jena, Germany) in combination with ZEISS Laser Slit Lamp 532s (Carl Zeiss, Jena, Germany). Only laser spots with visible formation of vaporisation bubbles were included in this study.

### Bone marrow transplantation

Bone marrow transplantation experiments were carried out as previously described [22]. In brief, a total of 12 recipient control and 14 *Irf8* knockout mice were head-shielded and lethally irradiated (RS2000 irradiator, Rad Source, Kanas, USA) in two independent experiments. Meanwhile, bone marrow cells (BMCs) were collected from the tibias and femurs of CAG-mRFP1 mice and resuspended in phosphate-buffered saline (PBS). The recipient mice were intravenously injected with 3 × 10^6^ BMCs via the tail vein. Nine weeks after bone marrow transplantation, the efficiency of reconstitution was assessed by flow cytometry which will be explained below.

### *In vivo* characterisation and analysis

Fundus morphology, retinal structure and physiological function were investigated using fundus photography, fundus fluorescein angiography (FFA), optical coherence tomography (OCT) and electroretinography (ERG) as previously described [32]. Fundus photography, FFA and OCT were performed using a Micron III retinal microscope (Phoenix Technology Group, Pleasanton, CA, USA) and the StreamPix software (Norpix Inc., Montreal, Canada). For FFA, 10% sodium fluorescein (Alcon, Freiburg, Germany) was diluted to a concentration of 50 μL/mL in 0.9% sodium chloride for injection (VWR, Leuven, Belgium) and administered intraperitoneally (2 μL/g). Ninety seconds after dye injection, the angiograms were recorded. For quantification of CNV size, hyperfluorescent areas in early-phase angiograms were measured in pixels using ImageJ. Image-guided OCT was performed using the OCT2 scan head. In OCT images, the thickness of the inner nuclear layer (INL) (200 pixels from the optic nerve head) was measured using ImageJ (https://imagej.nih.gov/ij/). For ERG, mice were dark-adapted overnight and anaesthetized by intraperitoneal injection of ketamine hydrochloride (66.8 mg/kg) and xylazine (12.76 mg/kg). ERG signals were amplified, recorded and analysed automatically using Ganzfeld Q450 (Roland-Consult, Brandenburg, Germany) with the integrated software developed by Prof. Dr. rer. nat. Michael Bach (Eye Center, University of Freiburg, Germany).

### Immunohistochemistry and imaging

After intracardiac perfusion with PBS and 4% paraformaldehyde (PFA), eyes were fixated in 4% PFA for 45 min at room temperature and processed to RPE-choroidal-scleral and retinal flat mounts. After incubation in PBST/BSA blocking buffer overnight, the flat mounts were incubated with primary antibodies against collagen type IV (1:500, AB769, Merck Millipore, Darmstadt, Germany), Iba1 (1:500, #019-19741, Wako, Neuss, Germany) or alpha smooth muscle actin (SMA, 1:500, ab5694, Abcam, Cambridge, UK) for two nights at 4 °C, followed by incubation with Alexa Fluor® 568 or 647-conjugated secondary antibodies overnight at 4 °C (1:500, Life technologies, Eugene, OR, USA). Eyes of *Irf8*-VENUS mice were fixated in 4% PFA for 1 h and incubated in 10%, 20% and 30% sucrose for 24 h each prior to embedding in Tissue-Tek O.C.T. compound (Sakura, Aplphen aan den Rijn, The Netherlands). Seven micrometre-thick cryosections were cut using a cryostat (Leica CM1950, Leica, Nussloch, Germany). Following blocking in Ultra V block for 10 min at room temperature, the sections were incubated with primary antibodies against ßIII tubulin (1:500, ab18207, Abcam, Cambridge, UK), collagen type IV (1:1000, ab6586, Abcam, Cambridge, UK), Ceh-10 homeo domain containing homolog (CHX10, 1:200, ab16141, Abcam, Cambridge, UK), GFP (1:500, 600-101-215, ROCKLAND, Limerick, PA, USA), glial fibrillary acidic protein (GFAP, 1:500, 087A1005RE, Fremont, CA, USA) or Iba1 (1:500) for 60 min with corresponding Alexa Fluor® 568-conjugated secondary antibodies (1:500, Life technologies, Eugene, OR, USA). Nuclei were counterstained with 4,6-diamidino-2-phenylindole (DAPI). Stainings were imaged using the Nano Zoomer S60 digital slide scanner (Hamamatsu, Herrsching am Ammersee, Germany) and analysed with NDP viewer software (Hamamatsu, Herrsching am Ammersee, Germany) or with a confocal laser scanning microscope (Zeiss LSM 510 or Leica TCS SP8 or Olympus FV1000), Zen software (Carl Zeiss, Jena, Germany), LAS X software (Leica, Nussloch, Germany) or Fluoview FV1000 (Olympus, Tokyo, Japan). For a detailed list of antibodies used, see supplementary table 2.

### Three-dimensional reconstruction of retinal microglia

Imaging for 3D reconstruction was performed using a Zeiss LSM 510 confocal laser scanning microscope with a 20× objective, 3× zoom and 1024 × 1024 pixel resolution. The interval thickness of the z-stacks was set to 1.0 μm. The morphology of retinal microglia in the inner plexiform layer (IPL) and outer plexiform layer (OPL) was determined by a three-dimensional reconstruction using the filament mode of IMARIS software (Bitplane, Zurich, Switzerland). Three cells per layer and mouse were reconstructed and analysed.

### Fluorescence-activated cell sorting

Following transcardial perfusion with 1× PBS and enucleation, eyes were dissected in ice-cold 1× PBS to isolate the retinae of *Irf8*^*+*/+^*Cx3cr*1^GFP/+^ *or Irf8*^-/-^*Cx3cr*1^GFP/+^. For the lasered mice, the central parts (70%) of the retinae were used for FACS while the peripheral parts were omitted. After tissue homogenisation and filtration through a 50-μm cell strainer (Sysmex, Goerlitz, Germany), dead cell exclusion was performed by incubation with fixable viability dye 780 (1:1000, 65-0865-14, eBioscience, Waltham, MA, USA). Anti-CD16/CD32 (Fc) receptor (1:200, 553142, BD Biosciences, Heidelberg, Germany) was used to avoid unspecific binding. Following staining with anti-CD45 (1:200, 103133, BioLegend, San Diego, CA, USA), anti-CD11b (1:200, 17-0112-83, eBioscience, Waltham, MA, USA), anti-Ly6C (1:200, 560593, BD Bioscience, Heidelberg, Germany) and anti-Ly6G (1:200, 560601, BD Biosciences, Heidelberg, Germany) for 20 min at 4 °C, retinal microglia characterised as CD45^low^CD11b^+^*Cx3cr1*^GFP/+^Ly6C^-^Ly6G^-^ were analysed and sorted into RNA stabilisation reagent (QIAGEN, Hilden, Germany) using a MoFlo Astrios EQ High Speed Cell Sorter (Beckman Coulter, Munich, Germany). For RNA sequencing, an average number of 10,000 retinal microglia per sample was obtained from pooling 3 to 4 mice. Flow cytometric purification of wild-type retinal and brain microglia together with bone marrow–derived monocytes was described before [59]. Data are available under the GSE accession number GSE160845. For a detailed list of antibodies used, see supplementary table 2.

### RNA extraction

RNA extraction, RNA library preparation and RNA sequencing were performed in collaboration with the Genomics Core Facility "KFB-Center of Excellence for Fluorescent Bioanalytics" (University of Regensburg, Germany). RNA extraction was performed according to manufacturer’s instructions using the RNeasy Plus Mini Kit (QIAGEN, Hilden, Germany). After pelleting the sample by centrifugation, the RNA stabilisation reagent was removed and replaced by RLT Plus buffer for lysing retinal microglia. Genomic DNA was removed selectively and efficiently by using gDNA Eliminator spin columns for RNA purification. After adding Ethanol to the flow-through, the sample was applied to an RNeasy MinElute spin column to collect RNA. Finally, after washing the column, total purified RNA was eluted in RNase-free water. The quality and integrity of total RNA was assessed with a Agilent 2100 Bioanalyser in combination with the RNA 6000 Pico LabChip Kit (Agilent, Palo Alto, CA, USA).

### RNA sequencing

First-strand cDNA was generated using SMARTer Ultra Low Input RNA Kit for Sequencing v4 (Clontech Laboratories, Inc., Mountain View, CA, USA). Double-standed cDNA was amplified with LD PCR and purified with AMPure XP beads. Library preparation was constructed conforming to the Illumina Nextera XT Sample Preparation Guide (Illumina, San Diego, CA, USA). In brief, 150 pg of input cDNA was tagmented via Nextera XT transposome. The products were purified and amplified with a limited-cycle PCR program to construct sequencing libraries. The libraries were quantified with the KAPA SYBR FAST ABI Prism Library Quantification Kit (Kapa Biosystems, Wobum, MA, USA). Equimolar amounts of each library were pooled for cluster generation on the cBot using the Illumina TruSeq SR Cluster Kit v3. The sequencing run was performed on a HiSeq1000 instrument with TruSeq SBS Kit v3 according to the Illumina HiSeq 1000 System User Guide. Illumina image analysis and base calling were recorded in library base call format (.bcl) and further converted to Fastq files via the CASAVA1.8.2 software.

### RNA sequencing data analysis

Quality control and transcriptome profiling including reads mapping, annotation, quantification and normalisation were performed by GenXPro (GenXPro, Frankfurt, Germany). Briefly, FastQC was performed to assess sequencing quality. After removing reads containing adapter sequences and duplicate reads via cutadapt software (GitHub, San Francisco, CA, USA) and FastUniq, the filtered reads were mapped to the mouse genome from ENSEMBL (https://www.ensembl.org/Mus_musculus/Info/Index) using bowtie2. The transcripts were functionally annotated with gene transfer format file version 90, quantified using HTSeq and normalised as transcripts per kilobase million (TPM) via DESeq2. Differential gene expression analysis with threshold (log2 fold change greater than 1.5 or less than − 1.5, *p* < 0.05, TPM ≥ 100 in at least one of the two compared groups) was performed using DESeq2. Data was visualised using RStudio (v1.2.1335) and R (v3.5.3). Volcano plots were created using the *ggplot2* package, and Gene Ontology (GO) analysis was performed using R with the *clusterProfiler 3.10.1* package [62].

### Protein analysis

Protein was extracted from the choroid and the retinae using RIPA buffer (#R2078, Sigma Aldrich) containing protease inhibitor (cOmplete, Mini; edta-FREE Protease inhibitor Cocktail, Roche Diagnostics, Manheim, Germany) and phosphatase inhibitors (PhosSTOP, Roche Diagnostics, Manheim, Germany), respectively, for preservation. Total protein concentration for each sample was measured with the Pierce^TM^ Bicinchoninic Acid Protein Assay Kit (Thermo Fisher Scienticis, Inc., Rockland, IL, USA).

### Statistical analysis

Statistical analysis was performed using GraphPad Prism v6 (La Jolla, USA) as follows: an unpaired *t* test was applied if the normality was given by the Kolmogorov-Smirnov test. Otherwise, the Mann–Whitney *U* test was used. Difference with significance was defined as *p* < 0.05.

## Results

### *IRF8* is the most abundantly expressed member of the IRF family in retinal microglia

**Fig. 1:**
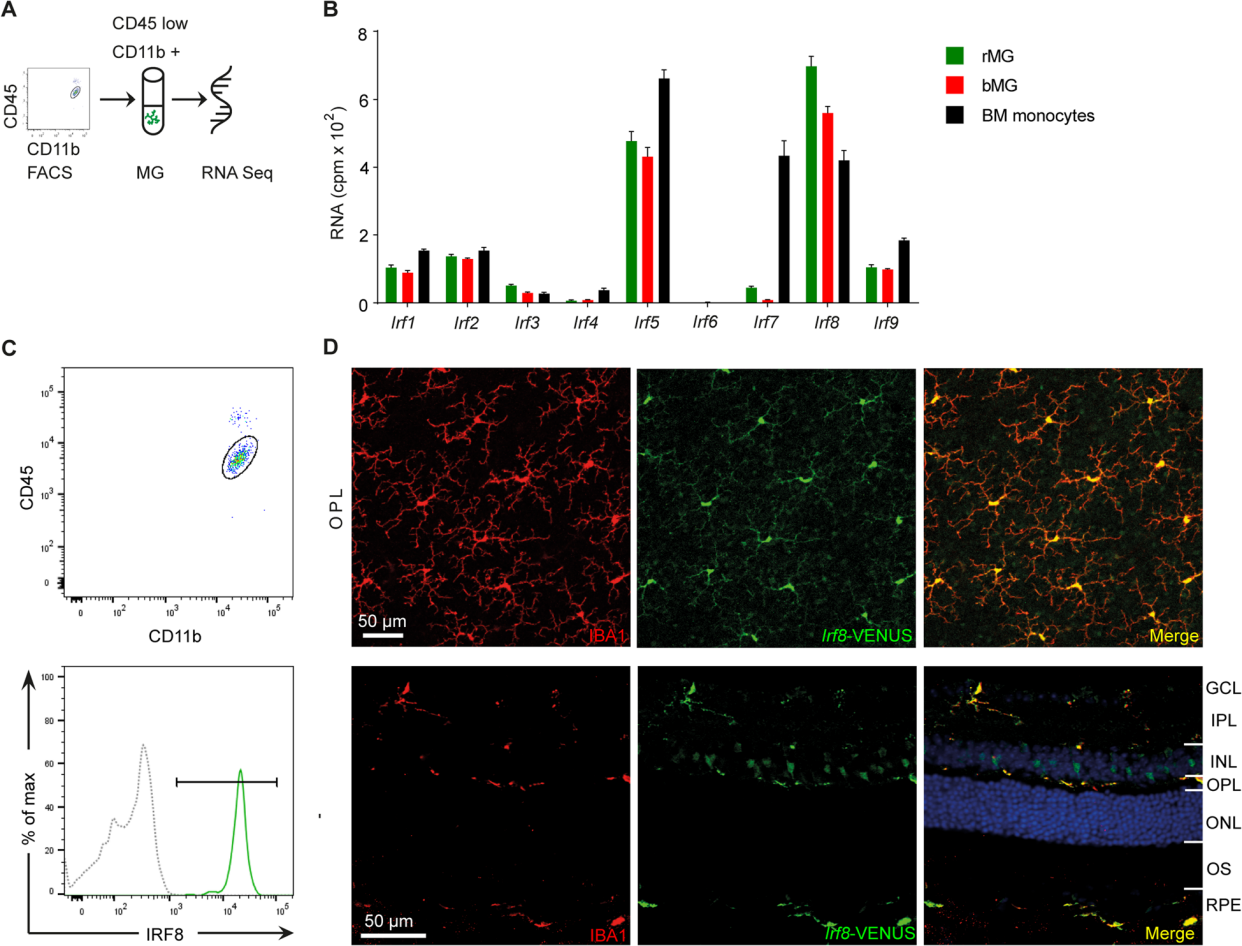
*Irf8* is predominantly expressed in retinal microglia (MG) and blood monocytes. **A** Flow chart of retinal MG RNA sequencing. **B** Differential expression of IRF family members in retinal MG (rMG), brain MG (bMG) and bone marrow (BM) monocytes. The gene expression profile was generated from four CD45^lo^CD11b^+^Ly6C^-^Ly6G^-^ rMG samples, four CD45^lo^CD11b^+^Ly6C^-^Ly6G^-^ bMG samples and four CD45^+^CD11b^+^Ssc^lo^CD115^+^Ly6C^+^ bone marrow (BM) monocytes. **C** Flow cytometry analysis of *Irf8*-VENUS expression in CD45^lo^CD11b^+^ retinal MG (98.7% ± 0.5, green solid line) in comparison with a negative control (grey dotted line). **D** Immunohistochemistry of the retinal flat mounts (upper panel) and cryosections (lower panel) from *Irf8*-VENUS mice reveal that all IBA1+ (red) rMG express *Irf8*-VENUS (green)

The interferon regulatory factor (IRF) family is critical for the development, maturation and function of myeloid cells [40]. Using flow cytometry and RNA sequencing, we first determined the expression levels of different IRF family members in adult retinal microglia (rMG), brain microglia (bMG) and bone marrow (BM) monocytes (Fig. 1A, B). In general, genes belonging to the IRF family were expressed at different levels in rMG, bMG and BM monocytes (Fig. 1B). Compared with other IRF family members, *Irf5* and *Irf8* exhibited the highest expression levels in rMG, bMG and BM monocytes. In the retina, *Irf8* emerged as the most prominent IRF member in rMG. Interestingly, the expression of *Irf8* in rMG exceeded the expression in bMG and BM monocytes indicating a distinct function of *Irf8* for rMG. To validate *Irf8* expression in adult rMG, we next analysed *Irf8*-VENUS reporter mice by flow cytometry and immunohistochemistry. Flow cytometry analysis revealed a strong VENUS expression (98.7% ± 0.5%) in CD45^lo^CD11b^+^ rMG in the steady state (Fig. 1C). In line with this finding, immunofluorescence analysis of retinal flat mounts and cryosections confirmed that IRF8 is mostly expressed in IBA1-positive microglial cells in the steady state (Fig. 1D and Supplementary Figure 1C). Since expression of Irf family members may change in response to stress and inflammation, we next assessed the expression levels of all IRF family members (1–9) and of common MG signature genes, such as *Tmem119* and *P2ry12*, in retinal MG in 2–3 months old *Cx3cr1*^GFP/+^ mice in the steady state and upon MG activation in the laser-induced CNV model. While the expression of the signature genes *P2ry12* and Tmem119 decreased in retinal MG after laser injury, activation markers such as *Cd74* were increased, as reported before [59]. However, most members of the IRF family were expressed at the same level in the rMG in the context of laser-induced inflammation compared with controls. Only *Irf2* and *Irf5* were slightly downregulated in retinal MG, whereas *Irf7* was modestly upregulated. Notably, the expression of *Irf8* in retinal MG remained stable in the laser-induced CNV model compared with controls (data not shown).

### Deficiency of *Irf8* has a substantial impact on morphology, distribution and transcriptional activity of retinal MG in the steady state

**Fig. 2:**
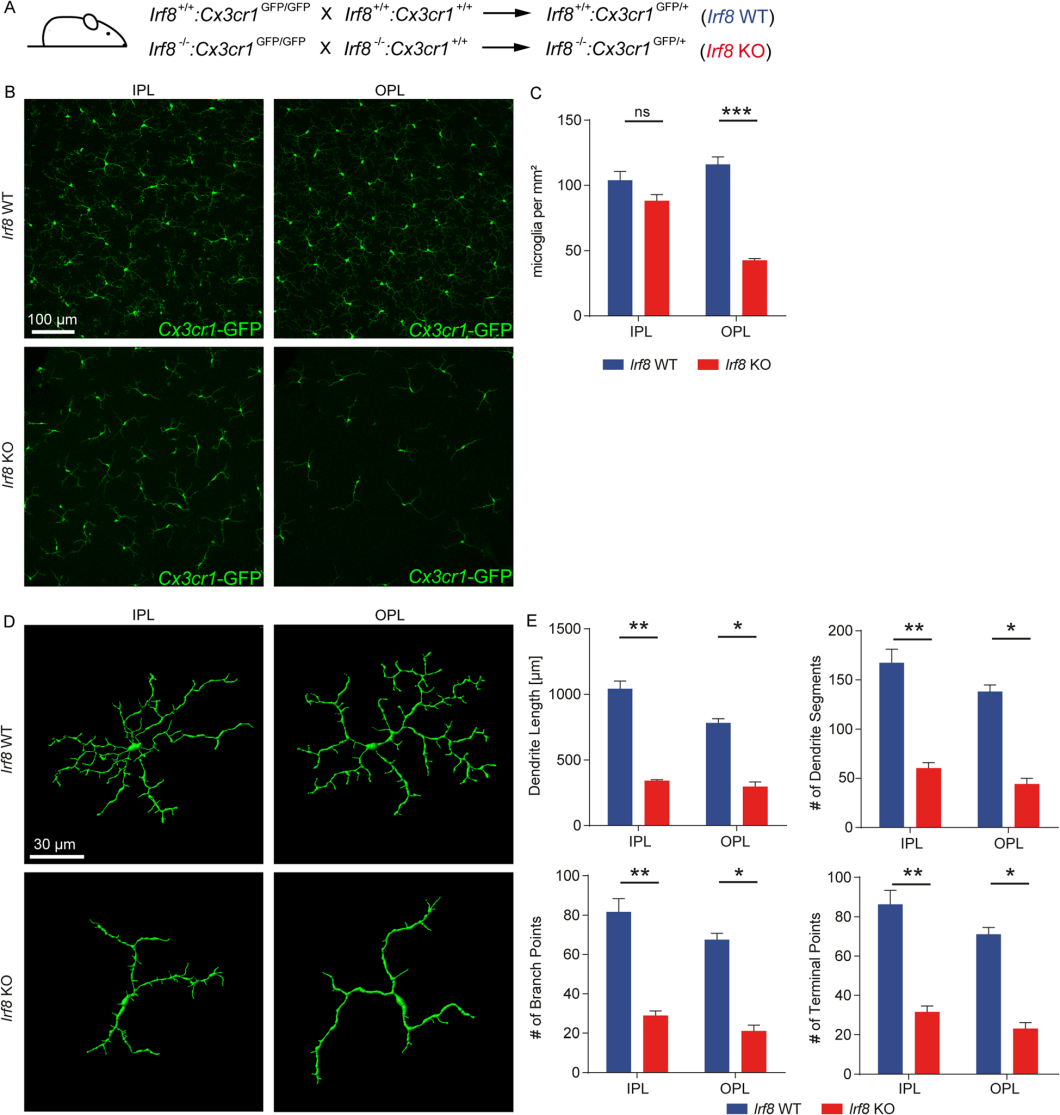
*Irf8* deficiency alters retinal MG distribution, morphology and transcriptional profile. **A** Breeding scheme. **B** *Irf8* KO mice demonstrate reduced density of *Cx3cr1-*GFP^+^ (green) retinal MG. Quantitative analysis are shown in (C). Pictures are representative for *n* = 5 mice in each group. **C** Quantification in microglial numbers in the inner plexiform (IPL) and outer plexiform layer (OPL). *n* = 5 mice were analysed per group. Data are shown as mean ± SEM. **D** 3D reconstruction of retinal MG by IMARIS reveals that *Irf8* deficiency alters morphology of retinal MG, resulting in shorter total length of dendrites, less dendrite segments and attenuated branching. **E** Quantitative analysis of retinal morphology. Four to six cells were reconstructed per mouse per IPL and OPL separately. Statistics were performed with the mean values per mouse (*n* = 6–7 in the *Irf8* WT and *n* = 3–4 in the *Irf8* KO group). Data are shown as mean ± SEM

To assess the role of IRF8 in distribution and cell morphology of rMG in the adult situation, we next examined retinal flat mounts from 8-week-old *Irf8* knockout (KO) mice by immunofluorescence microscopy (Fig. 2A,B). *Irf8* KO mice revealed a strikingly altered rMG distribution and morphology compared with wild-type (WT) animals, characterised by slightly reduced MG numbers in the inner plexiform layer (IPL, *Irf8* WT: 104 ± 7 cells/mm^2^, *Irf8* KO: 88 ± 5 cells/mm^2^, *p* = 0.09) and highly significant decrease of cell numbers in the outer plexiform layer (OPL, *Irf8* WT: 116 ± 6 cells/mm^2^; *Irf8* KO: 43 ± 1 cells/mm^2^, *p* < 0.0001, Fig. 2B,C). Quantitative morphometric analysis using IMARIS revealed a severely altered morphology of retinal microglia in *Irf8* KO mice, including significantly shorter length of dendrites (IPL: *Irf8* KO: 356.5 ± 14.3 μm, *Irf8* WT 1043.0 ± 59.4 μm, *p* = 0.009; OPL: *Irf8* KO: 297.7 ± 35.6 μm, *Irf8* WT: 788.8 ± 26.1 μm, *p* = 0.01), and reduced number of dendrite segments (IPL: *Irf8* KO: 62 ± 4, *Irf8* WT: 167 ± 14, *p* = 0.009; OPL: *Irf8 KO*: 44 ± 6, *Irf8* WT: 139 ± 6, *p* = 0.01), branch points (IPL: *Irf8* KO: 30 ± 2, *Irf8* WT: 82 ± 7, , *p* = 0.009; OPL: *Irf8* KO: 21 ± 3, *Irf8* WT: 68 ± 3, *p* = 0.01) and terminal points (IPL: *Irf8* KO: 33 ± 2, *Irf8* WT: 86 ± 7, *p* = 0.009; OPL: *Irf8* KO: 23 ± 3, *Irf8* WT: 71 ± 3, *p* = 0.01, Fig. 2D,E) compared with controls. Having established a profoundly altered rMG distribution and phenotype in the adult situation, we next explored rMG cell numbers at earlier stages of postnatal development. Interestingly, reduced microglia cell numbers were already present in the neuroblast layer at postnatal day 1 (P1) and later in the OPL at P7 but only transiently in the IPL at P7 that could be compensated until adulthood (Suppl. Figure 2A, B). These findings suggest an impaired MG distribution specifically in the deeper layers of the retina which is already present shortly after birth and persists into adulthood.

**Fig. 3:**
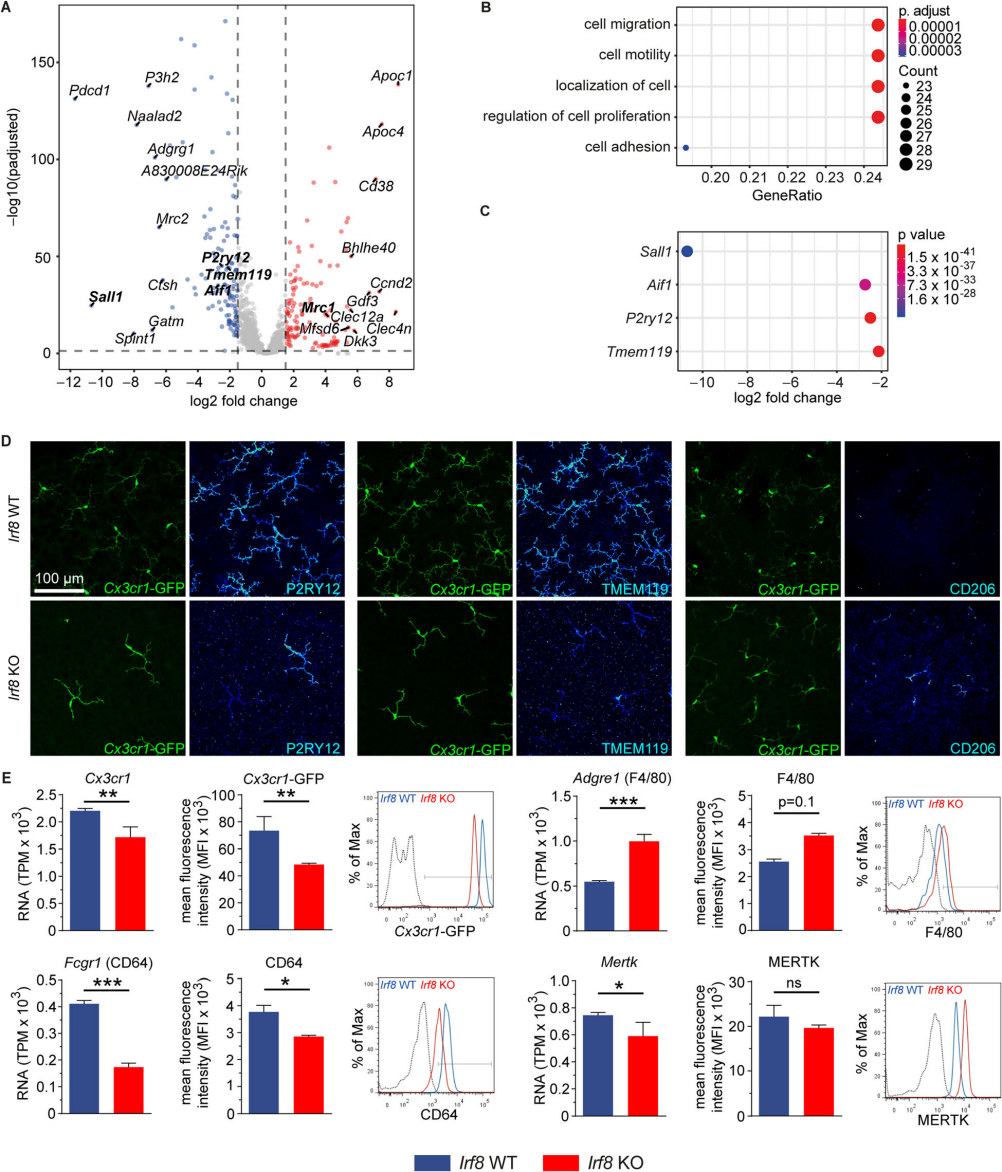
*Irf8* deficiency leads to expression loss of homeostatic signature genes. **A** Volcano plot of differentially expressed genes in *Irf8* KO retinal MG (*n* = 3) compared with control (*n* = 5). Significantly up- and downregulated genes are shown in red and blue, respectively. The top significantly up- and downregulated genes are labelled. **B** The top 5 downregulated GO clusters in *Irf8* KO retinal MG. Significance is represented as p.adjust, the size of each data circle indicates the number of genes involved in each enriched GO term. **C** Representative signature genes found to be highly expressed in competent retinal MG are significantly downregulated in the *Irf8* KO mice. **D** Immunohistochemistry of retinal flat mounts demonstrate a strong immunoreactivity for P2RY12 and TMEM119 shown as colour-coded signal intensity in *Irf8*-competent retinal MG that is reduced or absent in *Irf8* KO mice. The mannose receptor CD206 (encoded by *Mrc1*) is absent under homeostatic conditions but detectable under *Irf8*-deficient conditions. **E** Myeloid expression levels shown as transcripts per million (TPM) and analogue surface marker expression, as determined by flow cytometry, of *Irf8* WT (blue) and *Irf8* KO (red) mice, expressed as mean fluorescence intensity (MFI) (left). Representative histograms are shown (right) including fluorescence minus one controls (grey line). Six mice per group were analysed for CX_3_CR1, CD64 and MERTK, three mice per group for F4/80. Data are shown as mean ± SEM

To explore the associated transcriptional changes in *Irf8*-deficient rMG, we next performed RNA sequencing (RNA-seq) of FACS-sorted rMG from adult *Irf8* KO mice and controls. We determined 277 differentially expressed genes (DEGs) of which 142 were significantly upregulated, and 135 genes were downregulated in *Irf8* KO microglia compared with microglia of control mice (Fig. 3A). Gene ontology (GO) cluster analysis revealed that most of these downregulated genes contribute to processes such as “*cell migration”* (GO:0016477, p.adj. < 1.3 × 10^-5^), “*cell motility”* (GO:0048870, p.adj. < 4.8 × 10^-5^), “*localization of cells”* (GO:0051674, p.adj. < 4.8 × 10^-5^), “*regulation of cell proliferation”* (GO:0032944, p.adj. < 3.5 × 10^-3^) and “*cell adhesion”* (GO:0007155, p.adj. < 7.7 × 10^-4^) suggesting a reduced migratory potential of retinal microglia in the *Irf8* KO mice compared with controls (Fig. 3B). Among the downregulated DEGs, we found numerous microglia signature genes essential for microglia homeostasis, including the *spalt like transcription factor 1* (*Sall1*, log2FC = − 10.69, − log10*p* = 24.57), *allograft inflammatory factor 1* (*Aif1* or *Iba1*, log2FC = − 2.73, − log10*p* = 35.04), *purinergic receptor P2Y, G-Protein couple 12* (*P2ry12,* log2FC = − 2.50, − log10*p* = 45.06) and *transmembrane 119* (*Tmem119*, log2FC = − 2.13, − log10*p* = 24.57) (Fig. 3A,C). In accordance with the RNA-seq data, we found a strong immunoreactivity for TMEM119 and P2RY12 in *Irf8*-competent rMG cells, which was almost absent in *Irf8*-deficient rMG. Conversely, we found an increased immunoreactivity for the mannose receptor (CD206, *Mrc1*) in *Irf8*-deficient rMG, consistent with and further supporting the RNA-seq results (Fig. 3D). In addition, flow cytometry analysis of homeostatic CD45^+^CD11b^+^ rMG confirmed our RNA-seq results showing a reduced but still detectable protein expression of CX_3_CR1 and CD64 in *Irf8*-deficient rMG compared with controls with a trend towards lower MERTK and higher F4/80 expression as reported before in bMG [44] (Fig. 3E).

Taken together these results strongly suggest that IRF8 plays a critical role in maintaining the distribution, morphology and homeostasis of retinal microglia and has a substantial impact on the transcriptional activity of rMG in the steady state.

### *IRF8* is not required for the development of the retinal structure, vasculature and function

Having established the quantitative and qualitative changes in *Irf8*-deficient rMG, we next investigated whether *Irf8*-deficiency influences retinal structure and electroretinal function during steady state. To this end, we examined the retina of adult *Irf8-*deficient and control mice using color fundus photography (CF), fluorescein angiography (FA), optical coherence tomography (OCT), electroretinography (ERG) and immunohistochemical staining of retinal flat mounts for retinal vessels (Suppl. Figure 3).

In general, *Irf8* KO mice showed regular retinal structure, vasculature and function compared with controls. Qualitative assessment of retinal structure and vessels on CF and FA images of *Irf8-*deficient and control mice revealed no gross abnormalities, particularly no vascular dye leakage as an indicator of disturbed vascular architecture or spontaneous neovascularisation (Suppl. Figure 3A,B). In OCT images, the thickness of the inner nuclear layer (INL) and the outer nuclear layer (ONL) containing the photoreceptors (PR) was similar in *Irf8-*deficient mice (INL: 15.2 ± 0.5 μm, ONL: 69.8 ± 1.0 μm) compared with control animals (INL: 15.8 ± 0.4 μm, ONL: 72.3 ± 1.0 μm, Suppl. Figure 3C). ERG measurements demonstrated similar dark-adapted scotopic and light-adapted photopic responses in *Irf8* KO mice and controls (Suppl. Figure 3D). Specifically, no significant difference was detected between *Irf8* KO mice and controls with respect to scotopic a-waves emanating from rods, scotopic b-waves corresponding to depolarisation of bipolar cells, and photopic b-waves arising from cones. Immunohistochemical staining of retinal flat mounts revealed a regular retinal vasculature in *Irf8* KO mice, including equal numbers of arteries labelled by smooth muscle actin (SMA, *Irf8* WT 6.2 ± 0.4, *Irf8* KO: 5.9 ± 0.3 per animal) and major vessels stained with Isolectin-B_4_ (IB4, *Irf8* WT: 11.3 ± 0.5, Irf8 KO: 11.0 ± 0,5 per retinal per animal, Suppl. Figure 3E). Additionally, branch points in the central superficial vascular plexus (*Irf8* WT: 17.0 ± 2.7, *Irf8* KO 22.1 ± 2.4 per animal), central deep plexus (*Irf8* WT: 8.7 ± 4.7, *Irf8* KO: 74.0 ± 3.4 per animal), peripheral superficial plexus (*Irf8* WT: 29.0 ± 3.5, *Irf8* KO: 28.1 ± 2.0 per animal) and peripheral deep plexus (*Irf8* WT: 59.0 ± 5.1, *Irf8* KO: 63.9 ± 5.7 per animal) were similar between both groups (Suppl. Figure 3F).

Overall, these data show that *Irf8* is not essential for the development and maintenance of homeostatic retinal structure, vascular network and function. This is particularly surprising given the significant changes in rMG cell numbers in the *Irf8* KO mice during development and in the adult. Thus, the impaired retinal MG cell morphology and expression profile in otherwise unremarkable retinal homeostasis in *Irf8*-deficient mice provide a unique opportunity to investigate the role of retinal MG in the development of CNV.

### *Irf8* deficiency aggravates CNV formation

**Fig. 4:**
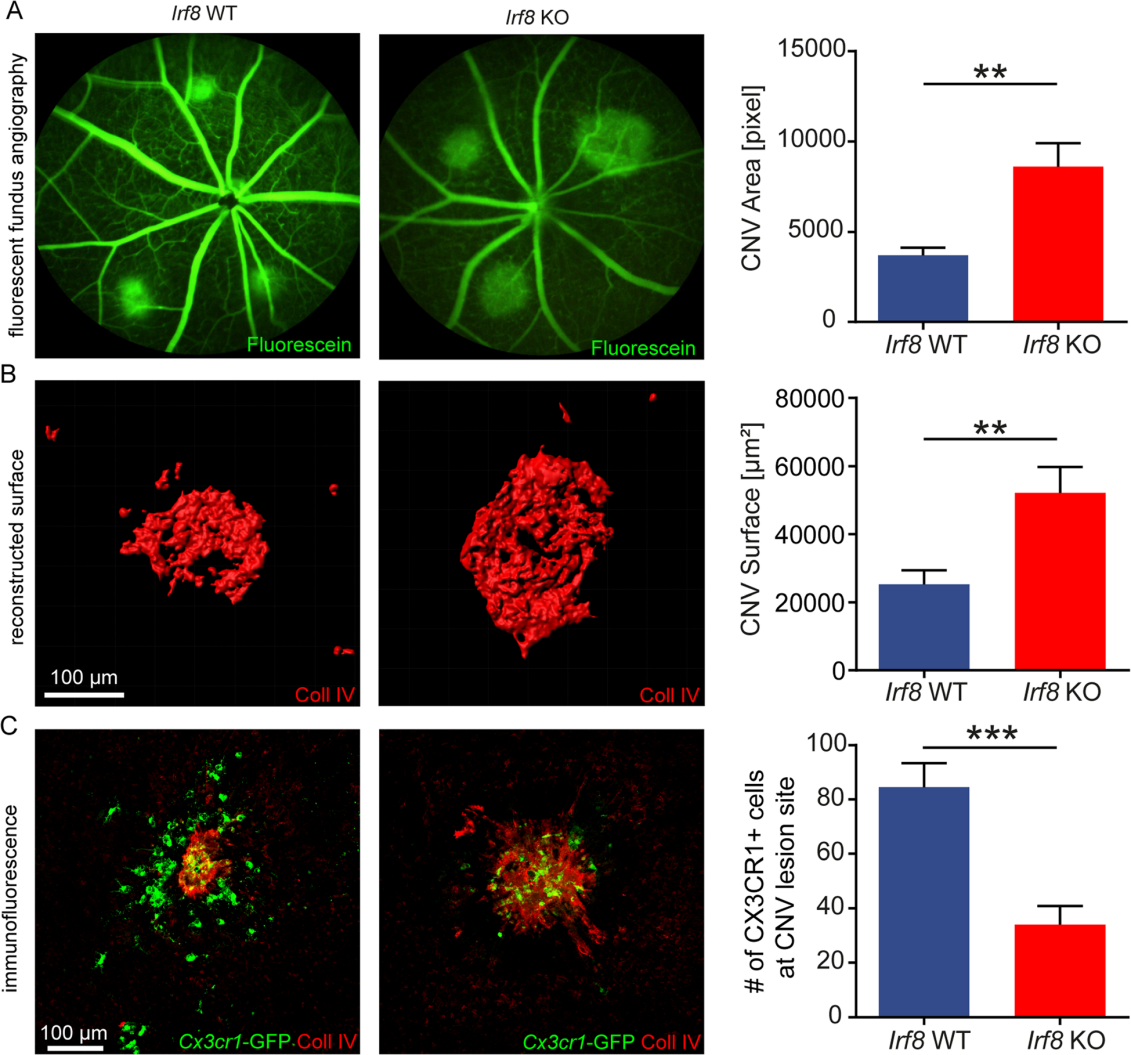
*Irf8*-deficiency aggravates CNV formation. **A** Fundus fluorescein angiography at day 7 following laser treatment demonstrates enlarged CNV lesions in *Irf8* KO mice (*n* = 11) compared with *Irf8* WT (*n* = 13). Data are presented as mean ± SEM. **B** 3D reconstruction of collagen type IV-labelled CNV lesions using IMARIS. IHC of RPE-choroid-scleral flat mounts confirms that *Irf8* deficiency (*n* = 11) increases CNV severity in comparison with control mice (*n* = 12). Data are presented as mean ± SEM. **C** Less *Cx3cr1*^GFP/+^ (green) cells around CNV lesions (red) at day 7 after laser treatment were observed in the *Irf8* KO mice (*n* = 6) compared with control mice (*n* = 7). Data are presented as mean ± SEM

Retinal MG change their phenotype and transcriptional profile after tissue injury and modulate the development of pathological CNV, which represents a hallmark of neovascular AMD [59]. To investigate the role of IRF8 in microglial cell activation after tissue injury and formation of CNV, we next studied *Irf8-*deficient and control mice in the laser-induced CNV model. Both *Irf8* KO and *Irf8* WT mice developed typical laser-induced CNV 7 days after laser photocoagulation visible, as hyperfluorescent lesions with clear demarcation in FA images (Fig. 4A). Quantification of hyperfluorescent CNV areas in angiograms revealed more than 2-fold larger CNV lesions in *Irf8* KO mice (8603 ± 1309 pixels per animal) compared with controls (3697 ± 425 pixels per animal, *p* < 0.005). Measurement of collagen type IV-labelled CNV area on RPE/choroidal flat mounts confirmed significantly enlarged CNV lesions in *Irf8* KO mice (52143 ± 7670 μm^2^) compared with *Irf8* WT (25203 ± 4156 μm^2^, *p* < 0.005, Fig. 4B). As expected, microscopic evaluation of RPE/choroidal flat mounts revealed that activated amoeboid *Cx3cr1-*GFP^+^ cells accumulate at CNV lesions in *Irf8* KO mice as well as in controls. The number of *Cx3cr1-*GFP^+^ cells around CNV lesions, however, was significantly decreased in *Irf8* KO mice (34 ± 7 cells per lesion per animal) compared with control animals (84.6 ± 8.8 cells per lesion per animal, *p* < 0.001, Fig. 4C). In addition, the number of rMG significantly increased in the IPL above CNV areas in controls, whereas no such increase was observed in *Irf8* KO mice, further pointing to a defect in rMG migration (Suppl. Figure 4).

Taken together, *Irf8*-deficient mice revealed reduced MG cell numbers suggesting an impaired MG migratory behaviour under physiological and pathological conditions which was associated with increased CNV lesion size in the laser-CNV model.

### Retinal microglia rather than infiltrating monocytes account for the larger CNV lesions in the *Irf8* KO mice

**Fig. 5:**
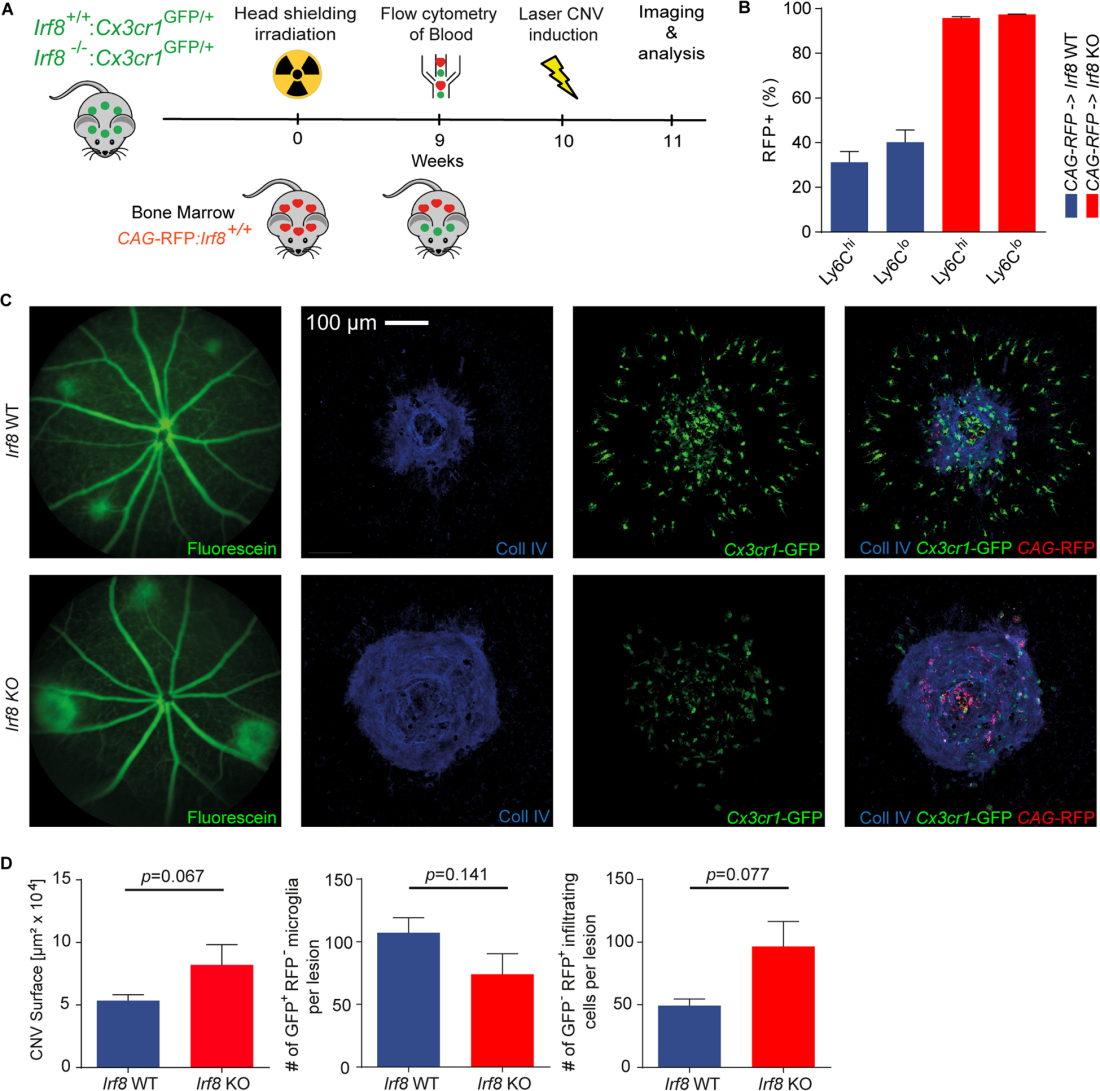
Bone marrow chimera experiments: reconstituted *Irf8*-deficient mice contain similar monocyte numbers as controls and reveal increased CNV lesion size. **A** Experimental setup. After head-shielded irradiation, bone marrow of *CAG*-RFP mice was transplanted intravenously to irradiated control (*Irf8*^+/+^:*Cx3cr1*^GFP/+^) and *Irf8*-deficient mice (*Irf8*^-/-^:*Cx3cr1*^GFP/+^) at the age of 8 weeks, respectively. Nine weeks after transplantation, flow cytometry was performed to check the reconstitution of blood cells. Ten weeks after transplantation, all mice underwent laser treatment to induce CNV. Analysis was performed at day 7 after laser induction. **B** After bone marrow transplantation, reconstitution of the blood cells was analyzed. Following head-shielded bone marrow transplantation, we observed a successful reconstitution of RFP^+^ peripheral CD45^+^CD11b^+^CD115^+^SSc^lo^Ly6C^hi/lo^ monocytes in *Irf8*-deficient animals compared with controls by using flow cytometry. *Irf8* KO (*n* = 6) and *Irf8* WT (*n* = 8) mice were used per group. Data are presented as mean ± SEM. **C**,**D** Following bone marrow transplantation, *Irf8*-deficient mice exhibit a 2-fold increase of laser-induced CNV compared with control mice, while the number of *Cx3cr1*^GFP/+^ GFP-positive and RFP-negative microglia at sites of CNV was similar in both groups. The number of reconstituted RFP-positive and GFP-negative blood-derived monocytes were increased in *Irf8*-deficient mice compared with controls. *Irf8* KO (*N* = 12) and *Irf8* WT (*n* = 14) mice were used per group. Data are presented as mean ± SEM

Since *Cx3cr1* is expressed in retinal microglia and infiltrating monocyte-derived macrophages from the blood [26], the observed *Cx3cr1*^GFP/+^ positive cells around CNV lesions could belong to both cell populations. However, *Irf8*-deficient mice are characterized by a low number of peripheral monocytes, which suggests that very few monocytes from the blood infiltrated the CNV lesion (Terry et al, 2015 PMID: 25277331). To investigate the influence of peripheral monocytes in our model, we next performed bone marrow transplantation experiments with bone marrow from CAG-RFP reporter animals to restore the peripheral monocyte pool in *Irf8*-deficient mice with *Irf8* potent monocytes (Fig. 5A). Following head-shielded bone marrow transplantation, we observed a successful reconstitution of RFP^+^ *Irf8*-potent peripheral monocytes in *Irf8*-deficient animals compared with controls by using flow cytometry (*Irf8* KO: 95.78 ± 0.59% (RFP^+^Ly6C^hi^), 97.42 ± 0.14% (RFP^+^Ly6C^lo^), *Irf8* WT: 31.19 ± 4.85% (RFP^+^Ly6C^hi^), 40.24 ± 5.5% (RFP^+^Ly6C^lo^), Fig. 5B). The observed higher recombination efficiency of Ly6C^hi^ and Ly6C^lo^ monocytes in *Irf8* KO is likely due to the initially low abundance of these cells and their respective progenitors in *Irf8* KO mice. Of note, due to the head shielding, the recipients’ bone marrow in the skull is still active and not substituted by the donor cells that could explain the comparably lower recombination efficiency in *Irf8* WT mice (Mildner et al, 2007 PMID: 18026096). Interestingly, the reconstituted *Irf8*-deficient animals still demonstrated increased CNV lesion size that was approximately twice as large compared with reconstituted *Irf8* WT animals (*Irf8* KO 82,009 ± 16,242 μm^2^; *Irf8* WT 53,386 ± 4793 μm^2^, *p* = 0.06) which was associated with slightly reduced numbers of RFP^-^GFP^+^ microglia at sites of CNV (*Irf8* KO 74 ± 16.5; *Irf8* WT 197.3 ± 12.1, *p* = 0.14) (Fig. 5C,D). The numbers of reconstituted RFP^+^GFP^-^ monocyte-derived macrophages, in contrast, were around twofold increased in *Irf8*-deficient mice compared with controls (*Irf8* KO 96.6 ± 20.1; *Irf8* WT 49.25 ± 5.3, *p* = 0.07) (Fig. 5D).

### Transcriptional profile of *Irf8* KO retinal microglia during CNV formation

**Fig. 6:**
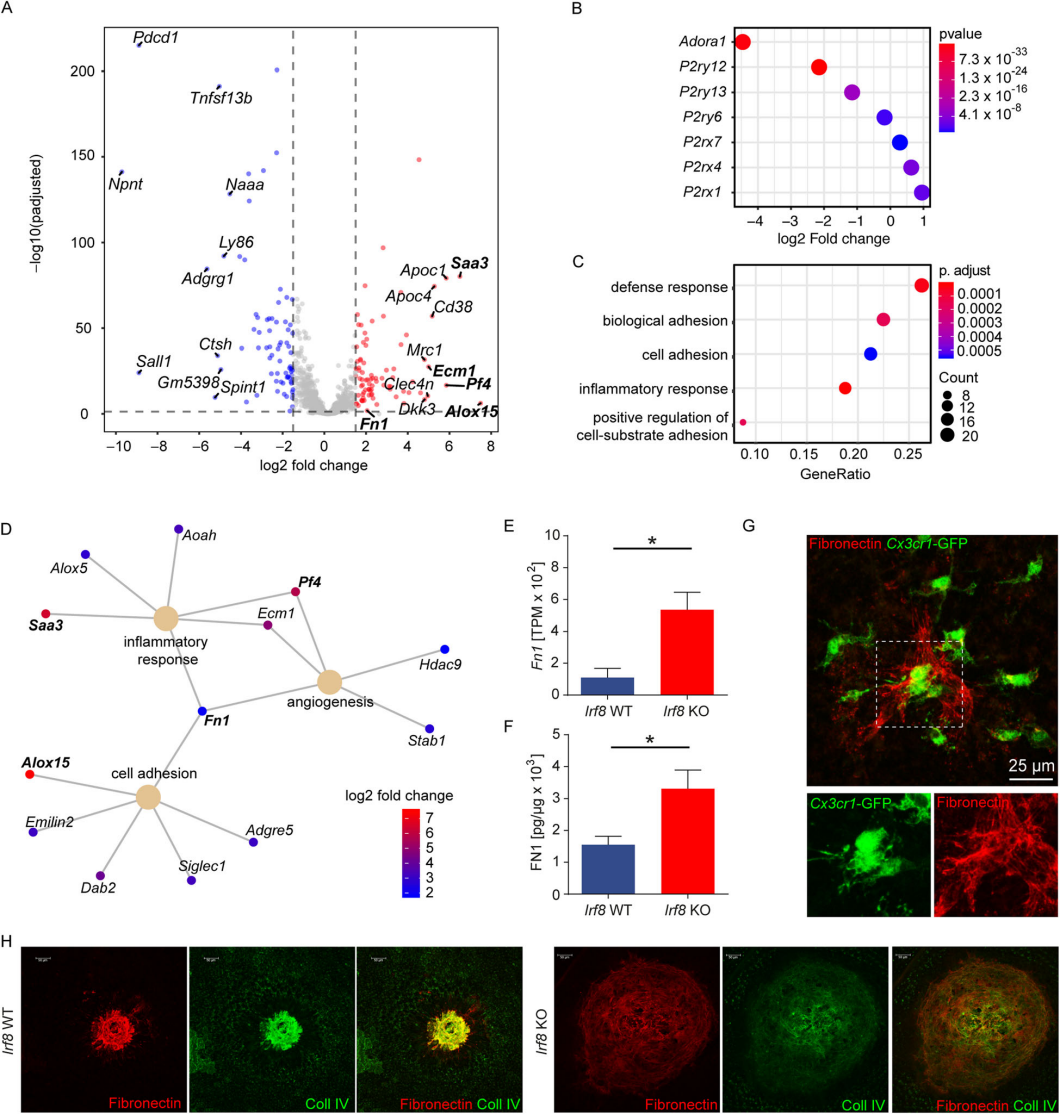
Transcriptional profiling of *Irf8* KO mice during CNV formation. **A** Volcano plot of differentially expressed genes in *Irf8* KO retinal MG (*n* = 4) compared with control (*n* = 5) after laser photocoagulation. Significantly up- and downregulated genes are shown in red and blue, respectively. The top significantly up- and downregulated genes are labelled. **B** The top 5 upregulated GO clusters in retinal MG of lasered *Irf8* KO mice. Significance is represented as p.adjust, the size of each data circle indicates the number of genes involved in each enriched GO term. **C** Differential expression of purinergic receptors in rMG from *Irf8* KO compared with *Irf8* WT mice. **D** Cnet-plot links Fibronectin (*Fn1*) to the GO terms *inflammatory response*, *angiogenesis* and *cell adhesion*. Genes written in bold belong to the top differentially upregulated genes (see **A**). **E** Expression of *Fn1* is significantly increased on transcriptional level in *Irf8*-deficient microglia, shown as transcripts per million (*Irf8* KO *N* = 4, *Irf8* WT *n* = 5). Data are presented as mean ± SEM. **F** Expression of FN1 is significantly increased on protein level in *Irf8*-deficient microglia, measured by ELISA. *N* = 5 mice per group. Data are presented as mean ± SEM. **G** Expression of *Fn1* can be traced back to myeloid *Cx3cr1*-GFP^+^ cells at the boarder of the CNV lesion at day 7 following laser. **H** Fibronectin and Collagen IV are strongly associated in the CNV lesions in both *Irf8* WT and *Irf8* KO mice

In order to decipher the molecular mediators of enhanced CNV formation in *Irf8* KO mice, we next isolated CNV-associated rMG from *Irf8* KO and control mice by flow cytometry and analysed the cells using RNA-seq. In total, we found 84 genes that were differentially upregulated and 78 genes that were downregulated in *Irf8*-deficient microglia 7 days following laser injury. In line with the RNA-seq analysis under homeostatic conditions, we identified similar DEG that were down- or upregulated in *Irf8*-deficient microglia after tissue injury, such as *Sall1*, *P2yr12* and *Mrc1* (Fig. 6A). The downregulation of *P2ry12* in laser-treated *Irf8* KO mice prompted us to explore the expression of other purinergic receptors which are critical for MG cell activation and migration. Here, we found several other genes encoding purinergic receptors to be strongly downregulated in *Irf8*-deficient MG, such as *Adora1*, *P2ry12* and *P2ry13,* underlining the proposed migration defect upon laser injury (Fig. 6B). Furthermore, we analysed the expression of key M1 (*Cd86, H2-Ab1, Tlr2*) and M2 signature genes (*CD163, Mrc1*) in isolated CNV-associated MG in *Irf8*-deficient mice and control animals (Suppl. Figure 5). We found that common M1 markers such as *Cd86*, *H2-Ab1* and *Tlr2* were significantly downregulated in *Irf8-*deficient MG compared with *Irf8*-potent MG in the laser CNV model. On the other hand, some of the common M2 markers, such as *Cd163* and *Mrc1,* were significantly upregulated in *Irf8*-deficient retinal MG compared with *Irf8*-potent MG at sites of CNV suggesting a M1 to M2 polarization in *Irf8*-deficient MG compared with wild-type MG in the laser CNV model.

Next, we analysed the DEG that were upregulated in *Irf8*-deficient MG by GO cluster analysis and found an activation of biological processes such as “*defense response”* (GO:0006952, p.adj. < 8.4 × 10^-6^), “*biological adhesion”* (GO:0022610, p.adj. < 2.9 × 10^-4^), “*cell adhesion”* (GO:0007155, p.adj. < 6.1 × 10^-4^), “*inflammatory response”* (GO:0006954, p.adj. < 9.5 × 10^-6^) and “*positive regulation of cell substrate adhesion”* (GO:0010811, p.adj. < 1.9 × 10^-3^) in *Irf8-*deficient MG (Fig. 6C). A closer look at the DEG of three key GO clusters essential for CNV development, namely "*angiogenesis*", "*inflammatory response*" and "*cell adhesion*", revealed Fibronectin, a profibrotic mediator encoded by *Fn1*, as a prominent upregulated gene in *Irf8*-deficient MG linking all three biological processes (Fig. 6D). In addition, we found a marked increase in the expression of other pro-fibrotic factors such as *Fgf2* and *Spp1* and a subtle increase in the expression of *Tgfb1* in CNV-associated retinal MG in *Irf8*-deficient mice compared with control mice (*data not shown*). Consistent with the increased number of *Fn1* transcripts in *Irf8*-deficient MG, we found significantly increased FN1 protein levels in the RPE/choroid of *Irf8*-deficient mice (3314 ± 587.1 pg/μg protein) compared with controls (1550 ± 268.5 pg/μg protein, *p* < 0.05) using ELISA on tissue lysates (Fig. 6E, F). Immunohistochemical studies showed that FN1 expression was restricted to the area of CNV lesions and expressed by microglia at sites of CNV (Fig. 6G). In addition to the enlarged collagen IV-positive CNV lesion described above, *Irf8*-deficient mice showed a markedly enlarged FN1-positive CNV lesion compared with WT mice, indicating increased fibrosis (Fig. 6H).

Taken together, these studies show that *Irf8*-deficient microglia exhibit a significantly altered expression profile in the laser CNV model with downregulated migratory genes, such as purinergic receptors, and upregulated pro-fibrotic factors, such as *Fn1*.

## Discussion

The interferon regulatory factor 8 (IRF8) is an essential transcription factor for the development, maturation and homeostasis of microglia (MG) in the brain and other tissue macrophages [19, 21, 27]. However, the role of IRF8 for retinal MG (rMG) during homeostasis and neovascular eye disease has not been elucidated so far. In this study, we show that *Irf8* is essential for a mature rMG gene expression profile and influences MG morphology, migration and the response to pathological neovascularisation.

Our results show that *Irf8* is strongly expressed in rMG in the steady state compared with other IRF family members. The expression of *Irf8* in rMG was even substantially higher than in brain MG (bMG), suggesting a distinct and tissue-specific function of *Irf8* in microglia of the retina. Morphological analysis revealed that *Irf8*-deficient mice exhibited an overall reduced branching of rMG as well as a decreased MG cell number specifically in the outer plexiform layer, which was already observed during postnatal development. These findings recapitulate findings in the brain [21, 27, 39, 44] and point to a migratory defect that is already present during postnatal retinal layering and maintained into adulthood. To gain further insight into the molecular changes in *Irf8*-deficient rMG, we isolated rMG by flow cytometry and analysed their transcriptional profile by RNA sequencing. Our analysis revealed significant transcriptional differences between *Irf8*-potent and *Irf8*-deficient microglia, which is consistent with several *in vitro* studies [25, 40]. We found that *Sall1* was the most downregulated MG signature gene in the retina of *Irf8-*deficient mice. This is of particular functional importance since SALL1 is a critical transcription factor for maintaining the homeostatic gene expression pattern of MG and thus regulates, for example, the expression of MG signature genes such as *Aif1, P2ry12* and *Tmem119* [6, 33, 55]. Accordingly, we found *Tmem119* and *P2ry12* as significantly reduced in *Irf8*-deficient rMG recapitulating the findings from human brain microglia which suggest a dependence of P2RY12 expression on IRF8 signalling [5]. Notably, because SALL1 is not exclusively expressed by microglia but also by astrocytes and oligodendrocytes [8, 38, 48], non-cell autonomous effects of this gene cannot be excluded. Further, single-cell RNA-seq (scRNA-Seq) of conditional *Irf8* KO mice (*Fcgr1*-Cre:*Irf8*^fl/fl^) revealed a strong downregulation of *Sall1*, *Aif1*, *Tmem119* and *P2ry12* in bMG while other genes, typically expressed in macrophages, like *Mrc1*, encoding the mannose receptor CD206, were upregulated [58]. In line with this study, we found a strong upregulation of *Mrc1* on transcriptional level and the encoded mannose receptor CD206 on protein level in *Irf8-*deficient rMG which is consistent with data from *Irf8*-deficient bMG [44]. In addition, other myeloid genes like *Fcgr1*, also known as CD64, were downregulated on RNA and protein level. These findings strongly support common transcriptional changes between retinal MG and brain MG under *Irf8*-deficient conditions, as shown before in a direct comparison of wild-type rMG and bMG by scRNA-seq [59]. Furthermore, it underscores that retinal microglial differentiation and maturation are highly dependent on a defined transcriptional program instructed by PU.1 [16], SALL1 [29] and IRF8.

To identify functionally related gene signatures, we performed a gene ontology (GO) enrichment analysis that revealed a downregulation of the clusters “*cell migration“*, “*cell motility”*, “*cell adhesion”* and “*regulation of cell proliferation”* in adult *Irf8*-deficient rMG in the steady state. The downregulation of these GO terms was consistent with the reduced number of rMG in the OPL in adult *Irf8* KO mice, supporting previous *in vitro* data describing IRF8 as an essential transcription factor for microglial motility and migration [28, 39]. The significant downregulation of genes relevant for cell migration could also explain the observed developmental phenotype and point to an impaired *Irf8*-dependent perception of guiding cues. During development, neurons constantly release guiding cues, such as purines and other extracellular nucleotides which guide microglia to colonise the developing outer retina [1, 37]. *Irf8*-dependent downregulation of sensors that detect these guiding cues, such as *Adora1* or *P2yr12*, may be responsible for the disruption of purinergic signalling in *Irf8*-deficient rMG, which attenuates their migratory capacity. Indeed, *in vitro* cultivated brain MG lacking *Irf8* had a strongly reduced phosphorylation of AKT after ATP treatment, thereby diminishing the ATP-mediated signalling pathways of P2RY12 [40]. This hypothesis is supported by *in vitro* studies showing a diminished entry of rMG into retinal explant cultures following an interruption of purinergic signalling [37]. Furthermore, proliferation of myeloid cells relies on IRF8 signalling [61] which could explain the insufficient compensatory expansion of MG in the OPL and contribute to the niche-dependent phenotype with reduced microglial density especially in the OPL in adult *Irf8*-deficient mice.

Despite the aforementioned significant changes in MG cell density and transcriptional profile, *Irf8*-deficient mice exhibited a normal retinal structure, vascular supply, and physiological function. This is particularly surprising as retinal MG are in close contact with retinal vessels, especially during development, and are known to shape the mature retinal vasculature [9, 13, 16, 18]. This suggests that *Irf8*-deficient MG are functionally sufficient to accompany physiological retinal development and that minor developmental disturbances, which we cannot completely rule out, can be compensated over time. The fact that *Irf8*-deficient microglia exhibit transcriptional changes that reduce their responsiveness while maintaining a normal retinal phenotype, provides an ideal setting to study the role of microglia in the development of choroidal neovascularisation, which is known to be associated with significant microglia activation and migration [35, 52, 59].

In the laser-induced CNV model, which mimics aspects of neovascular AMD, we found significantly increased CNV lesion size under *Irf8*-deficient conditions. At the same time, the overall numbers of *Irf8*-deficient *Cx3cr1*-GFP^+^ cells were significantly reduced around the lesions compared with wild type. In line with this finding, MG density in the IPL above CNV lesions was significantly decreased in *Irf8* KO mice compared with wild type. These observations may be mediated either by a migration defect caused by impaired purinergic signalling [45] or by reduced microglial proliferation of *Irf8*-deficient MG, or both, as discussed above. Interestingly, the present study shows that impaired migration and a lower number of MG in the vicinity of CNV are associated with a more severe CNV phenotype in *Irf8*-deficient mice. This finding is in contrast to previous studies showing that depletion of retinal or circulating myeloid cells by clodronate is associated with reduced CNV size suggesting an anti-angiogenic effect of myeloid cells on CNV [14, 34, 51]. On the other hand, however, our results are consistent with reports showing that increased accumulation of myeloid cells in neovascular lesions is associated with decreased CNV and that myeloid cells use FasL (CD95L) to inhibit CNV formation [2]. These seemingly contradictory contributions of myeloid cells to CNV development could be reconciled by the potentially different roles of resident MG and infiltrating monocyte-derived macrophages. To investigate the distinct roles of these cellular populations, this study exploits *Irf8*-deficient mice which are characterized by a near absence of circulating monocytes and, at the same time, altered but present resident retinal microglia. The enlarged CNV lesions in *Irf8*-deficient mice associated with reduced numbers of *Cx3cr1*-GFP^+^ resident microglia cells may thus be interpreted as a consequence of dysfunctional pro-fibrotic *Irf8*-deficient MG or as a result of insufficient numbers of CNV-suppressing microglia in *Irf8*-deficient animals. The latter may in turn indicate a general protective role of wild-type microglia which would be in line with previous studies [2, 46]. The hypothesis that deficient microglia substantially influence CNV size in *Irf8*-deficient mice is further supported by the head-shielded bone marrow transplantation experiments performed in this study, which showed enlarged CNV in *Irf8*-deficient animals compared with controls despite successful reconstitution of *Irf8-*potent peripheral monocytes. However, we cannot exclude the possibility that other *Irf8*-expressing cell types in the retina or from the blood contribute to the increased CNV lesion size in *Irf8*-deficient mice. Since *Irf8* expression in the retina was mainly restricted to retinal microglial cells, which represent the most numerous myeloid cell population in CNV, in contrast to a low number of infiltrating peripheral monocytes [59], we consider this possibility rather unlikely. Furthermore, heterozygous *Cx3cr1*^*GFP/+*^ expression could have an impact on myeloid cells and be associated with an inflammatory phenotype. However, since both *Irf8* wild-type and knockout mice in our study were consistently heterozygous for *Cx3cr1*, we consider this effect negligible and used the *Cx3cr1*^*GFP/+*^ line to visualise *Irf8* wild-type and knockout mice in a comparable manner, since other standard markers such as IBA1 are dysregulated and cannot be used [44].

To explore potential mechanisms in *Irf8*-deficient retinal MG contributing to CNV formation, we next performed RNA-sequencing on sorted retinal MG at sites of CNV.

Among others, we found that purinergic receptors, encoded by genes such as *P2ry12*, *P2ry13* and *Adora1*, were significantly downregulated in *Irf8* KO rMG after laser injury. Since these receptors are critical for the recognition of ATP released during tissue injury and thus control cell migration, their downregulation may be at least partly responsible for the reduced MG cell number at sites of CNV in *Irf8*-deficient mice [12, 23, 30]. This hypothesis is in line with *in vitro* work by Masuda et al. showing a downregulation of genes encoding purinergic receptors, including *P2ry12* and *P2rx4,* in *Irf8*-deficient brain microglia which was associated with a migration defect *in vitro* [39]. It is interesting to note that *Irf8*-deficient retinal microglia accumulating at CNV showed less M1 and more M2 signature gene expression compared with wild-type MG at CNV, suggesting increased M1 to M2 polarization in *Irf8*-deficient MG. This is of particular clinical interest as some studies have suggested that more M2-like macrophages, which are assumed to be pro-angiogenic, accumulate at the site of wet compared with dry AMD and that the pathological shift of macrophage polarization may contribute to the pathogenesis of CNV in neovascular AMD [7]. The expression signatures or myeloid cells at the lesion site are likely to be more complex than the aforementioned polarization state; however, it confirms an altered signature of a set of well-known and characterized markers. Furthermore, GO cluster analysis showed that *Irf8*-deficient rMG exhibit increased activity of biological processes that are critical for inflammation and cell adhesion, which points to an interaction of microglia with extracellular matrix components driving CNV formation. We hereby identified Fibronectin, encoded by the *Fn1* gene, as being significantly higher expressed in *Irf8* KO rMG and linking the GO terms "*cell adhesion"*, "*inflammatory response"* and "*angiogenesis"*. The immunohistochemical and ELISA analyses confirmed that the expression of Fibronectin protein was also significantly increased at sites of CNV, suggesting that *Irf8*-deficient MG or other cells affected by the loss of *Irf8* contribute to increased Fibronectin abundance at sites of CNV. Of note, collagen IV fibers were reported to depend on an established Fibronectin matrix directly co-localizing with Fibronectin fibers *in vitro* that could be functionally related to our observation of increased CNV lesion size promoted by higher Fibronectin expression in rMG [42, 43]. Furthermore, we found a marked increase in the expression of the pro-fibrotic factors *Fgf2* and *Spp1* and a subtle increase in the expression of *Tgfb1* in CNV-associated retinal MG in *Irf8*-deficient mice compared with control mice. Since FGF2 and SPP1 are important mediators of scarring and have been identified in human choroidal neovascular membranes [17, 52], overexpression of these factors in retinal MG may have further contributed to increased CNV lesion size in *Irf8*-deficient mice. This is of particular interest as pharmaceutical inhibition of SPP1 has been shown to modulate CNV formation and inhibition of FGF2 was associated with reduced CNV and subretinal fibrosis in a laser-induced mouse CNV model [3, 41, 52].

## Conclusions

In conclusion, this study identifies IRF8 as a critical mediator for the morphology, distribution and expression profile of retinal microglia, and for transformation to a reactive phenotype. The niche-dependent phenotype already present during postnatal development, the altered morphology and the disturbed rMG distribution did not lead to any impairment of retinal morphology and function in the steady state, but to enlarged lesions in the laser CNV model. This highlights the importance of IRF8 and retinal MG for the development of pathological neovascularisation in the eye and highlights the potential of immunomodulatory therapeutic interventions of rMG recruitment in retinal disease.

## Supplementary Information

**Additional file 1. **Supplemental figure 1 Irf8 is expressed predominantly in retinal MG and in some bipolar or Müller cells. No *Irf8*-VENUS expression could be detected co-localised with GFAP (A), βIII-Tubulin (B) and Collagen IV (E), indicating that IRF8 is not expressed in retinal astrocytes, ganglion cells or vessels. All IBA1^+^ cells exhibited a strong *Irf8*-VENUS signal (C) suggesting that all retinal MG express IRF8. Some CHX10^+^ cells could be co-localised with *Irf8*-VENUS expression (D) demonstrating that some bipolar cells or Müller cells express VENUS.

**Additional file 2.** Supplemental figure 2 Temporal and spatial distribution of retinal microglia during development. A Representative pictures showing the numbers of microglia per field of view in *Irf8* WT and *Irf8* KO mice in comparison between the ganglion cell and inner plexiform layer (GCL/IPL) and the developing neuroblast layer (NBL) or outer plexiform layer (OPL), respectively, at postnatal day (P) 1, P7 and in adult mice. B Quantification thereof. Data are presented as mean ± SEM.

**Additional file 3.** Supplemental figure 3 *Irf8* deficiency does not affect the retinal structure, function and vasculature. A-C Representative color fundus images (A), fluorescein angiography (B) and optical coherence tomography (OCT) images of *Irf8* WT and *Irf8* KO mice. C) Both *Irf8* WT (blue, n=12) and *Irf8* KO (red, n=12) mice displayed a regular retinal structure, a similar thickness of the inner nuclear layer and the outer retina at 100 and 200 μm from the optic nerve head in the optical coherence tomographs. Data are shown as mean ± SEM. ONH = Optic nerve head. D Electroretinography (ERG). No significant difference was found between the *Irf8* WT (blue, n=9) and *Irf8* KO (red, n=9) mice concerning the dark-adapted scotopic and light-adapted photopic ERG measurements at different flash intensities. Data are shown as mean ± SEM. E Staining against smooth muscle actin (SMA, red) reveals a comparable number of arteries (*Irf8* WT (n=5); *Irf8* KO (n=7)) and major vessels (*Irf8* WT (n=11); *Irf8* KO (n=13)) between both groups. Data are shown as mean ± SEM. F No significant differences in vessel branch formation in the superficial (upper panel) or deep plexus (lower panel) in the central or peripheral area of the retina could be observed, compared between *Irf8* WT (n=6) and *Irf8* KO (n=7). Data are presented as mean ± SEM.

**Additional file 4.** Supplemental figure 4 Spatial distribution of retinal microglia in CNV lesions. A Spatial distribution of microglia in the inner plexiform layer (IPL) in lasered and unlasered *Irf8* WT and *Irf8* KO mice. B Quantification of the numbers of microglia per field of view in the inner plexiform layer in lasered and unlasered *Irf8* WT and *Irf8* KO mice. Data are presented as mean ± SEM.

**Additional file 5.** Supplemental figure 5: Polarisation markers expressed by CNV-associated microglia. The M1 and M2 polarisation markers *Cd86*, *H2-Ab1*, *Tlr2*, *Cd163* and *Mrc1* (CD206) are shown as transcripts per million in comparison between *Irf8* WT and KO under CNV conditions.

**Additional file 6.** Supplemental table 1: List of primer sequences.

**Additional file 7.** Supplemental table 2: List of antibodies used for immunohistochemistry and flow cytometry.

## Data Availability

RNA sequencing data are available under the GSE accession number GSE160845 and GSE182504.

## Abbeviations

Adora1: Adenosine A1 receptor
Aif1: Allograft inflammatory factor 1
AMD: Age-related macular degeneration
ATP: Adenosine triphosphate
CNS: Central nervous system
CNV: Choroidal neovascularization
CSF1R: Colony stimulating factor 1 receptor
ELISA: Enzyme-linked immunosorbent assay
ERG: Electroretinography
FFA: Fundus fluorescein angiography
FN: Fibronectin
GO: Gene ontology
INL: Inner nuclear layer
IRF: Interferon regulatory factor
MG: Microglia
OCT: Optical coherence tomography
ONL: Outer nuclear layer
P2ry12: Purinergic receptor P2Y, G-Protein couple 12
PR: Photoreceptors
Sall1: Spalt-like transcription factor 1
Tmem119: Transmembrane 119
WT: Wild type
