# Supplementary figures and images for: The role of interferon regulatory factor 8 for retinal tissue homeostasis and development of choroidal neovascularisation
Source: J Neuroinflammation. 2021 Sep 20;18:215. doi: 10.1186/s12974-021-02230-y (PMC8454118; doi:10.1186/s12974-021-02230-y)

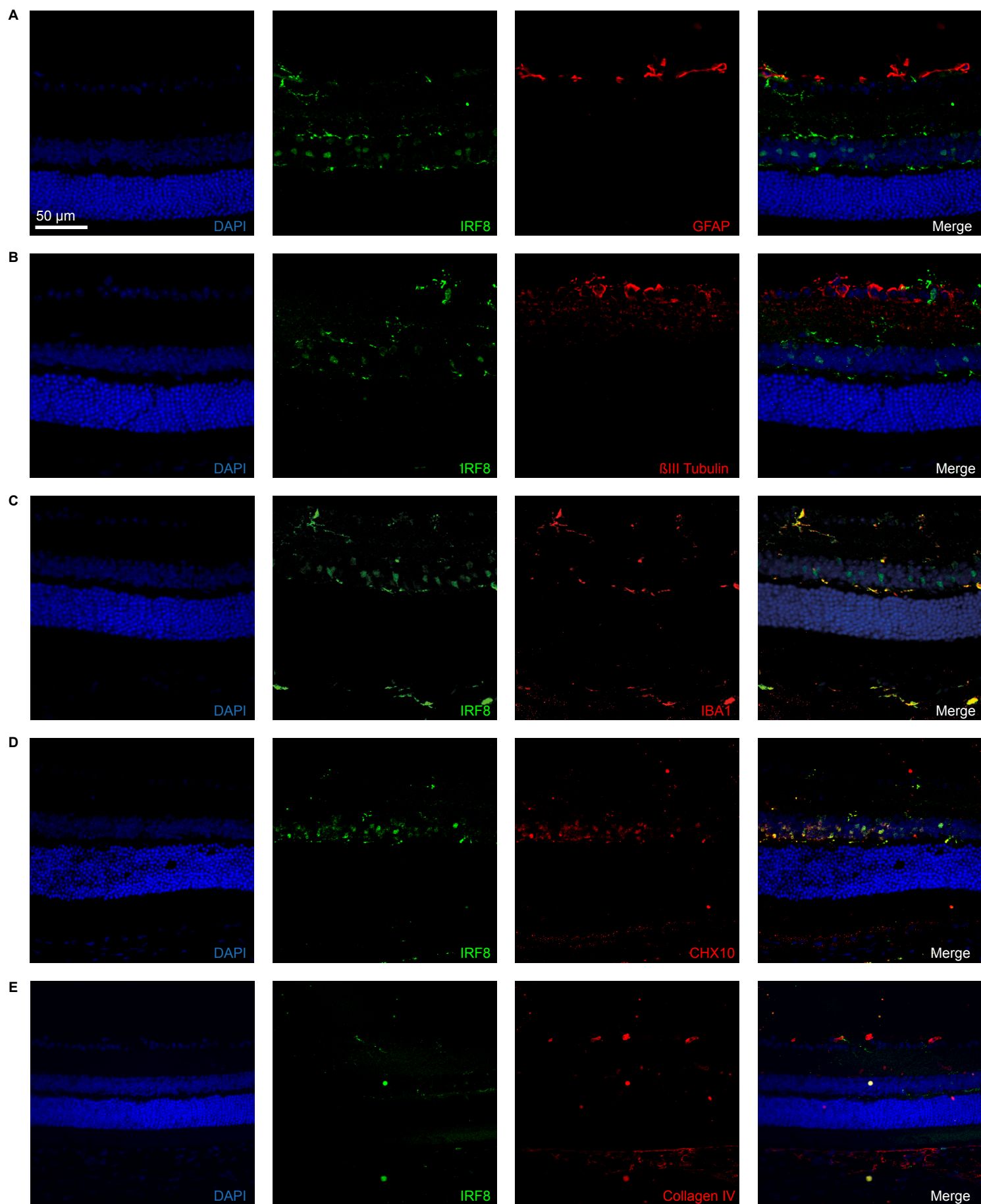

Suppl. Figure 1

Supplement: Supplementary file 1 — Additional file 1. Supplemental figure 1 Irf8 is expressed predominantly in retinal MG and in some bipolar or Müller cells. No Irf8-VENUS expression could be detected co-localised with GFAP (A), βIII-Tubulin (B) and Collagen IV (E), indicating that IRF8 is not expressed in retinal astrocytes, ganglion cells or vessels. All IBA1+ cells exhibited a strong Irf8-VENUS signal (C) suggesting that all retinal MG express IRF8. Some CHX10+ cells could be co-localised with Irf8-VENUS expression (D) demonstrating that some bipolar cells or Müller cells express VENUS. [file 12974_2021_2230_MOESM1_ESM.pdf]

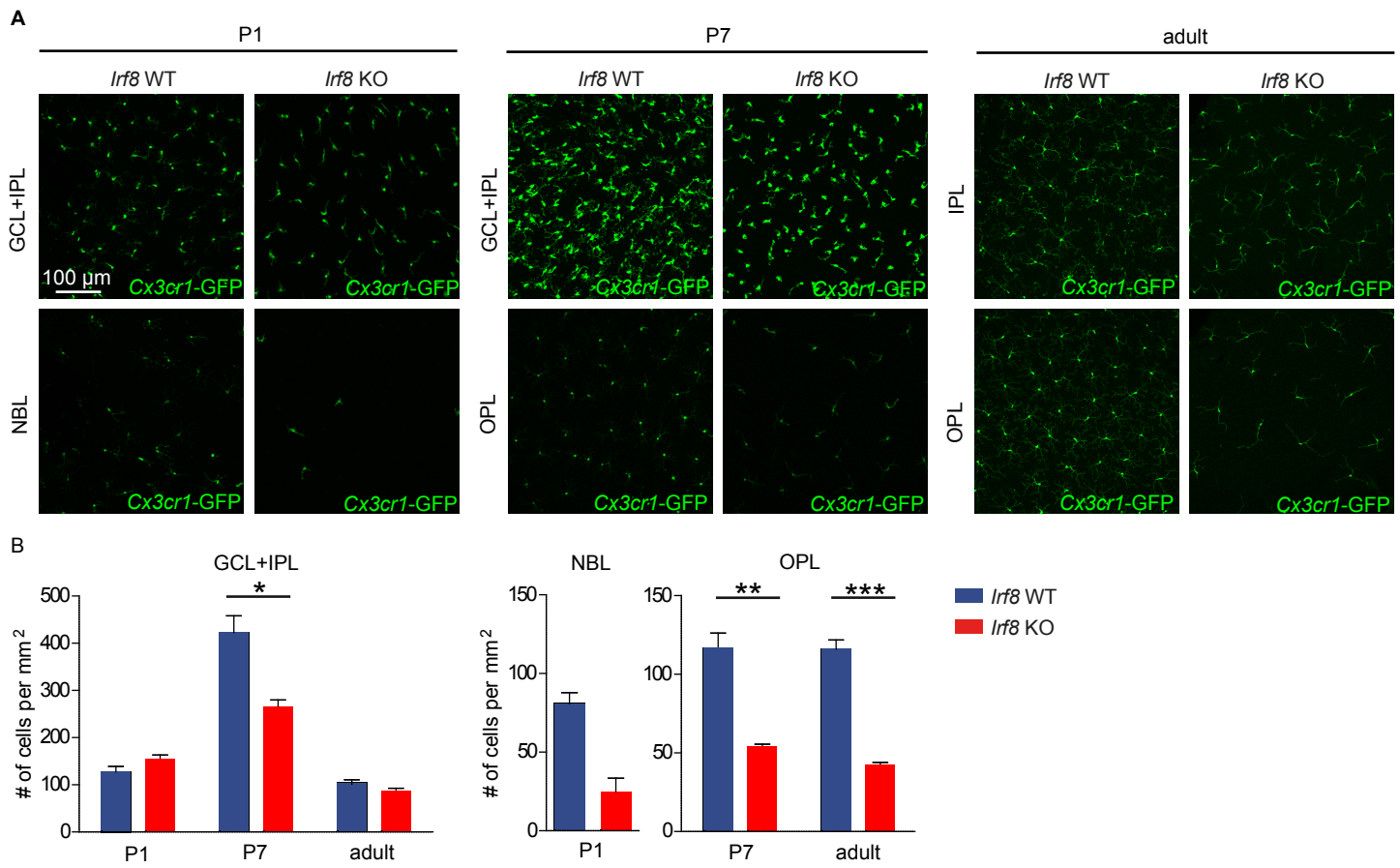

Supplement: Supplementary file 2 — Additional file 2. Supplemental figure 2 Temporal and spatial distribution of retinal microglia during development. A Representative pictures showing the numbers of microglia per field of view in Irf8 WT and Irf8 KO mice in comparison between the ganglion cell and inner plexiform layer (GCL/IPL) and the developing neuroblast layer (NBL) or outer plexiform layer (OPL), respectively, at postnatal day (P) 1, P7 and in adult mice. B Quantification thereof. Data are presented as mean ± SEM. [file 12974_2021_2230_MOESM2_ESM.pdf]

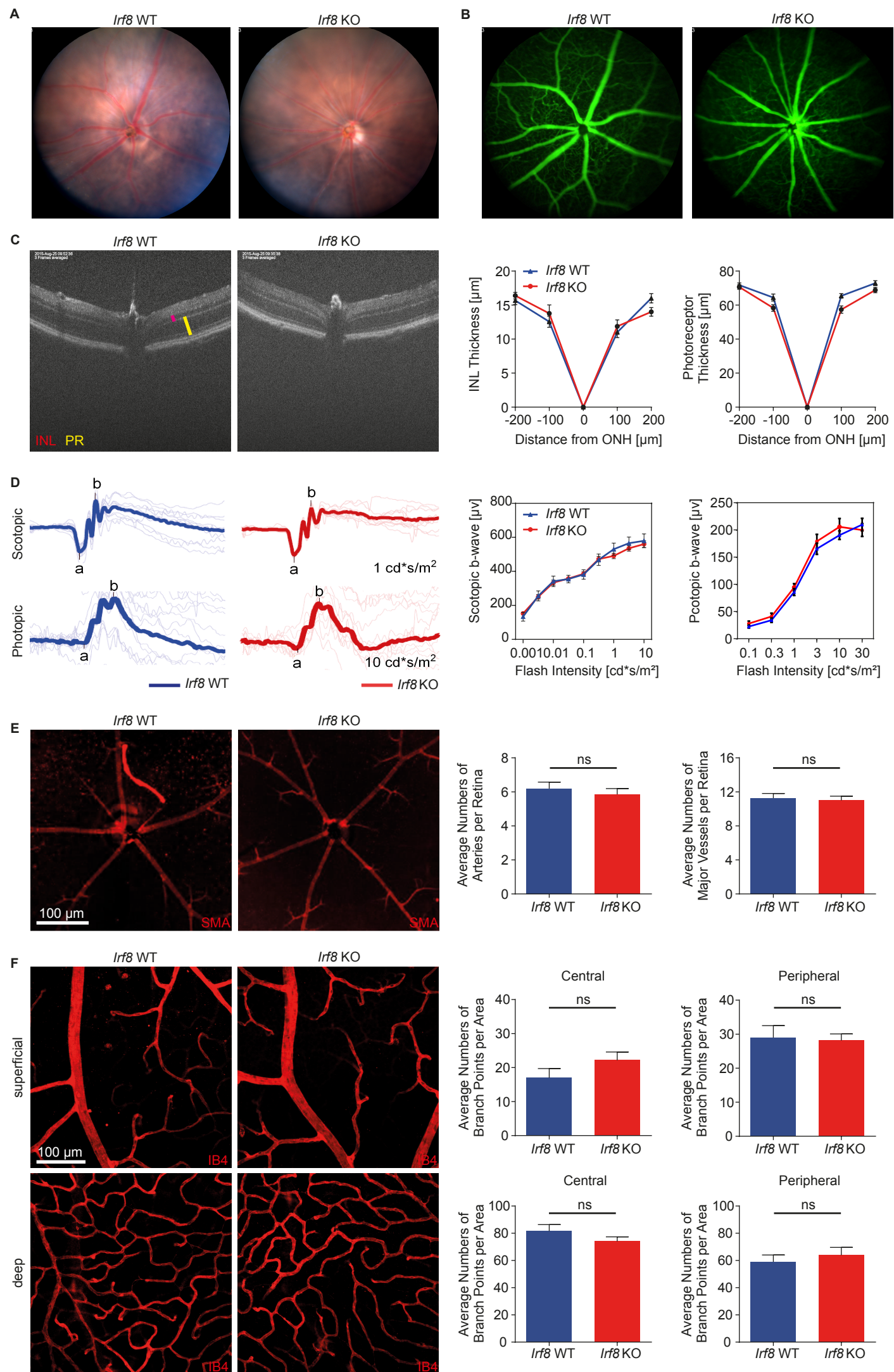

Supplement: Supplementary file 3 — Additional file 3. Supplemental figure 3 Irf8 deficiency does not affect the retinal structure, function and vasculature. A-C Representative color fundus images (A), fluorescein angiography (B) and optical coherence tomography (OCT) images of Irf8 WT and Irf8 KO mice. C) Both Irf8 WT (blue, n=12) and Irf8 KO (red, n=12) mice displayed a regular retinal structure, a similar thickness of the inner nuclear layer and the outer retina at 100 and 200 μm from the optic nerve head in the optical coherence tomographs. Data are shown as mean ± SEM. ONH = Optic nerve head. D Electroretinography (ERG). No significant difference was found between the Irf8 WT (blue, n=9) and Irf8 KO (red, n=9) mice concerning the dark-adapted scotopic and light-adapted photopic ERG measurements at different flash intensities. Data are shown as mean ± SEM. E Staining against smooth muscle actin (SMA, red) reveals a comparable number of arteries (Irf8 WT (n=5); Irf8 KO (n=7)) and major vessels (Irf8 WT (n=11); Irf8 KO (n=13)) between both groups. Data are shown as mean ± SEM. F No significant differences in vessel branch formation in the superficial (upper panel) or deep plexus (lower panel) in the central or peripheral area of the retina could be observed, compared between Irf8 WT (n=6) and Irf8 KO (n=7). Data are presented as mean ± SEM. [file 12974_2021_2230_MOESM3_ESM.pdf]

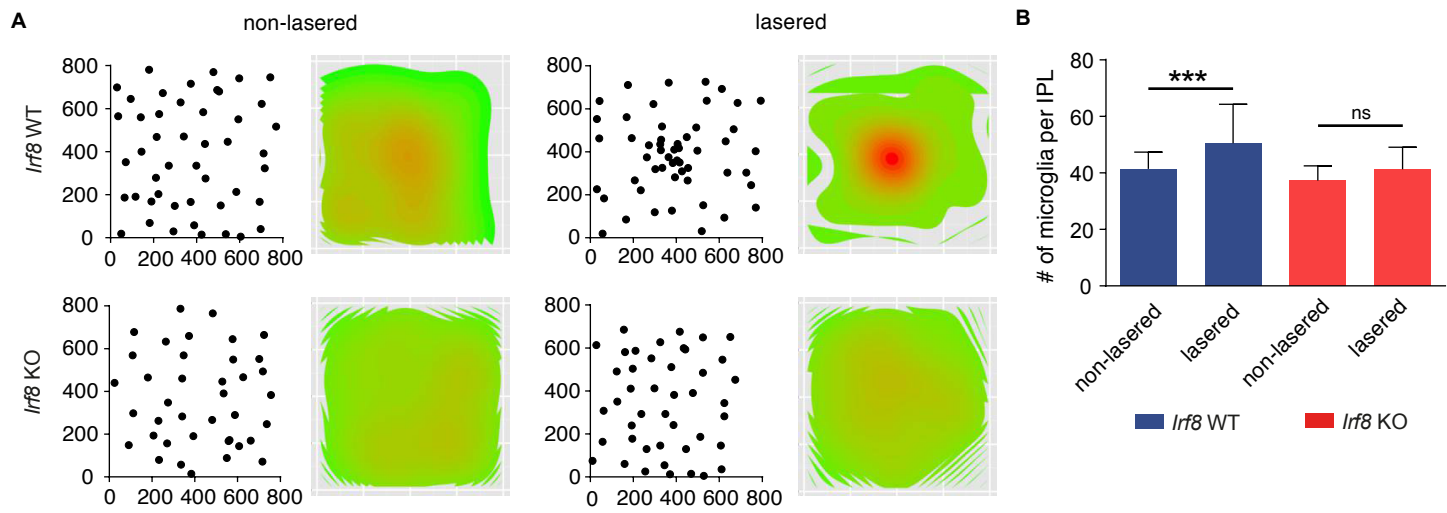

Supplement: Supplementary file 4 — Additional file 4. Supplemental figure 4 Spatial distribution of retinal microglia in CNV lesions. A Spatial distribution of microglia in the inner plexiform layer (IPL) in lasered and unlasered Irf8 WT and Irf8 KO mice. B Quantification of the numbers of microglia per field of view in the inner plexiform layer in lasered and unlasered Irf8 WT and Irf8 KO mice. Data are presented as mean ± SEM. [file 12974_2021_2230_MOESM4_ESM.pdf]

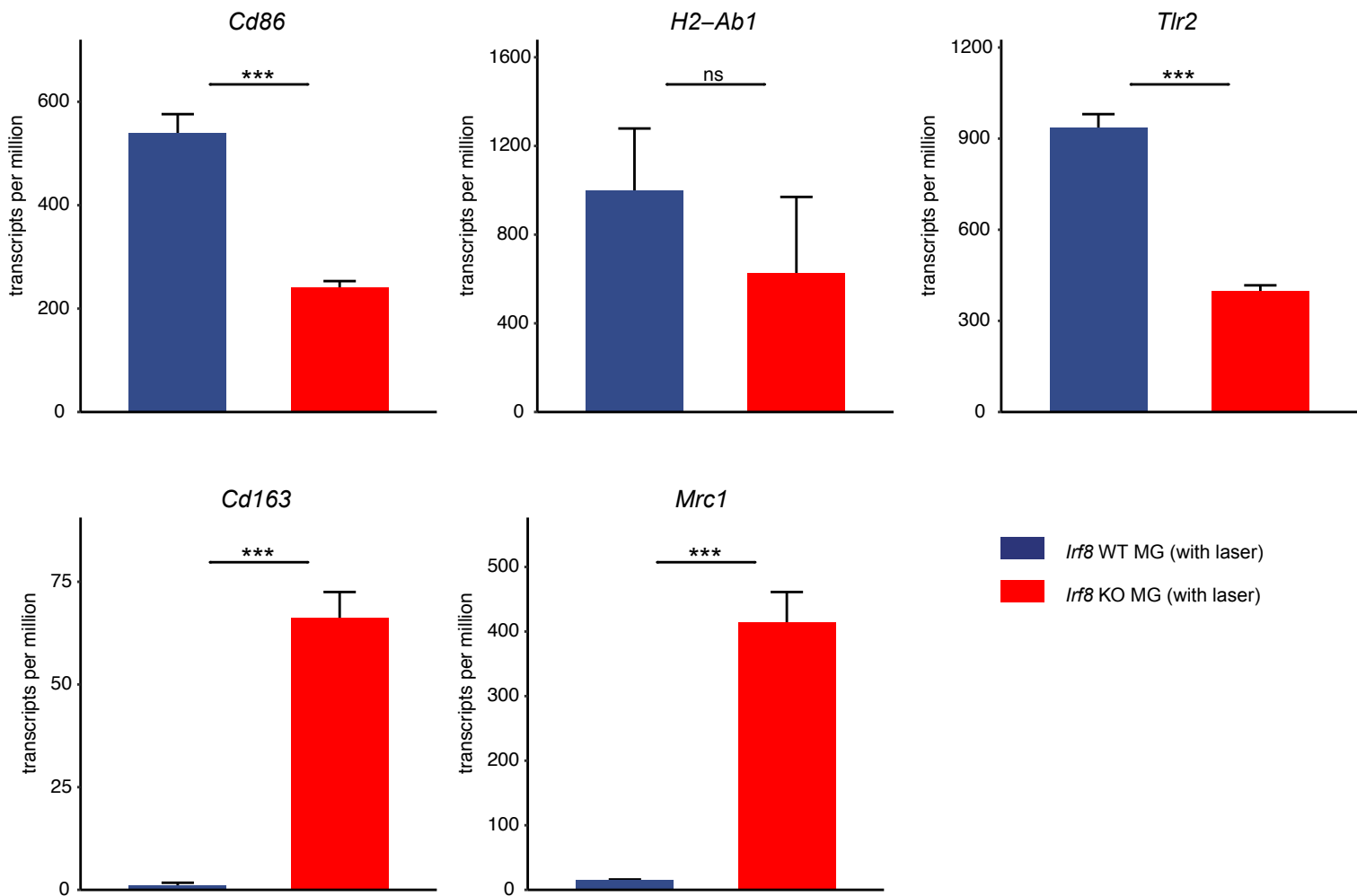

Suppl. Fig. 5

Supplement: Supplementary file 5 — Additional file 5. Supplemental figure 5: Polarisation markers expressed by CNV-associated microglia. The M1 and M2 polarisation markers Cd86, H2-Ab1, Tlr2, Cd163 and Mrc1 (CD206) are shown as transcripts per million in comparison between Irf8 WT and KO under CNV conditions. [file 12974_2021_2230_MOESM5_ESM.pdf]
