# Supplementary material for: The role of interferon regulatory factor 8 for retinal tissue homeostasis and development of choroidal neovascularisation
Source: J Neuroinflammation. 2021 Sep 20;18:215. doi: 10.1186/s12974-021-02230-y (PMC8454118; doi:10.1186/s12974-021-02230-y)
Supplement: Supplementary file 6 — Additional file 6. Supplemental table 1: List of primer sequences. [file 12974_2021_2230_MOESM6_ESM.pdf]

Primer	Sequence
IRF8_A:	CAT GGC ACT GGT CCA GAT GTC TTC C
IRF8_B:	CTT CCA GGG GAT ACG GAA CAT GGT
IRF8_C:	CGA AGG AGC AAA GCT GCT ATT GGC C
Cx3cr1-gfp-1:	TTC ACG TTC GGT CTG GTG GG
Cx3cr1-gfp-2a:	GCT GCA CTG TCC GGT TGT T
Cx3cr1-gfp-3:	GAT CAC TCT CGG CAT GGA CG
Irf8 _Venus PAC-1:	CAG TCC TAA GAC CCA GTG AAA AGC
Irf8 _Venus PAC-2:	ATA ACA TAT AGA CAA ACG CAC ACC

	Step	Temp	Time	go to	loop
	[#]	[°C]	[sek]		
	1	94	300	-	-
	2	94	30	-	-
IRF8	3	63	25	-	-
Cx3cr1-gfp	3	66	30	-	-
Irf8 _Venus	3	55	20	-	-
	4	72	60	2	33
	5	72	300	-	-
	6	4	∞	-	-

	mut	wt
IRF8	500	150
Cx3cr1-gfp	500	307
Irf8 _Venus	220	-

Supplemental Table 1
