# Supplementary material for: The role of interferon regulatory factor 8 for retinal tissue homeostasis and development of choroidal neovascularisation
Source: J Neuroinflammation. 2021 Sep 20;18:215. doi: 10.1186/s12974-021-02230-y (PMC8454118; doi:10.1186/s12974-021-02230-y)
Supplement: Supplementary file 7 — Additional file 7. Supplemental table 2: List of antibodies used for immunohistochemistry and flow cytometry. [file 12974_2021_2230_MOESM7_ESM.pdf]

Antibodies for Immunofluorescence	Company	Catalog Number
rabbit anti-Iba-1, polyclonal, 1:500 (cryo) or 1:1000 (flat-mount)	WAKO, Osaka, Japan	cat # 019-19741
rabbit anti-P2RY12, polyclonal, 1:500	AnaSpec, Fremont, USA	cat # AS-55043A
rabbit anti-Tmem119, polyclonal, 1:500	Synaptic Systems, Göttingen, Germany	cat # 400 002
goat anti-GFP, polyclonal, 1:500	Rockland Immunochemicals Inc., Gilbertsville, USA	cat # 600-106-215
Rabbit anti-mouse Collagen type IV, 1:1000	Abcam, Cambridge, UK	cat # ab6586
goat anti-Collagen type IV, 1:500	Merck Millipore, Darmstadt, Germany	cat # AB769
rabbit anti-alpha smooth muscle actin (SMA), 1:500	Abcam, Cambridge, UK	cat # ab5694
rabbit anti betaIII tubulin, 1:500	Abcam, Cambridge, UK	cat # ab18207
sheep anti-Ceh-0 homeo domain containing homolog (CHX10), 1:200	Abcam, Cambridge, UK	cat # ab16141
mouse anti-glial fibrillary acidic protein (GFAP), 1:500	Thermo Fisher Scientific Inc., USA	cat # MS-1376-R7
rat anti-F4/80 (clone BM8), monoclonal, 1:100	Abcam plc., UK	cat# ab16911
rabbit anti-Fibronectin, 1:100	Merck Millipore, Darmstadt, Germany	cat# AB2033
rat anti-CD206-Alexa Flour® 647 (clone MR5D3), monoclonal, 1:100	Bio-Rad Laboratories, UK	cat # MCA2235A647
goat anti-rabbit, Alexa Fluor 647®, polyclonal, 1:500	Thermo Fisher Scientific Inc., USA	cat # A-21244
Chicken anti-Rat-Alexa Fluor® 647	Thermo Fisher Scientific Inc., USA	cat # A-21472
Donkey anti-Goat-Alexa Fluor® 488	Thermo Fisher Scientific Inc., USA	cat # A-11055
Donkey anti-Goat-Alexa Fluor® 568	Thermo Fisher Scientific Inc., USA	cat # A-11057
Donkey anti-Sheep-Alexa Fluor® 568	Thermo Fisher Scientific Inc., USA	cat # A-21099
Donkey anti-Rabbit-Alexa Fluor® 568	Thermo Fisher Scientific Inc., USA	cat # A-10042
Donkey anti-Rabbit-Alexa Fluor® 647	Thermo Fisher Scientific Inc., USA	cat # A-31573

Antibodies for Flow Cytometry	Company	Catalog Number
Fc receptor blocking antibody CD16/CD32 (clone 2.4G2)	BD Pharmingen, BD Biosciences, Heidelberg, Germany	cat # 553141
rat anti-CD11b-BV421 (clone M1/70), monoclonal, 1:100	BioLegend, USA	cat # 101235
rat anti-CD11b-APC Cy7	BD Biosciences	cat # 557657
rat anti-CD11b-APC	Thermo Fisher Scientific Inc., USA	cat # 17-0112-83
rat anti-CD115-PE-Cy7 (AFS98), monoclonal, 1:100	Thermo Fisher Scientific Inc., USA	cat # 25-1152-82
rat anti-CD115-APC	Invitrogen	cat # 17-1152-82
mouse anti-CD45.2-PacB	Thermo Fisher Scientific Inc., USA	cat # 48-0454-82
rat anti-CD45-APC-e780 (30-F11), monoclonal, 1:100	Thermo Fisher Scientific Inc., USA	cat # 47-0451-82
rat anti-CD45-BV421 (30-F11), monoclonal, 1:100	BioLegend, USA	cat # 103134
rat anti-F4/80-PE-Cy7 (BM8), monoclonal, 1:100	Thermo Fisher Scientific Inc., USA	cat # 25-4801-82
mouse anti-CD64-Alexa647 (X54-5/7.1), monoclonal, 1:100	BioLegend, USA	cat # 139322
rat anti-Ly6C-PE Cy7	BD Pharmingen, BD Biosciences, Heidelberg, Germany	cat # 560593
rat anti-Ly6G-PE-Cy7 (clone 1A8), monoclonal, 1:100	BD Pharmingen, BD Biosciences, Heidelberg, Germany	cat # 560601
anti-CD45-BV421 (clone 30-F11), monoclonal, 1:100	BioLegend, USA	cat # 103134
rat anti-CD3-PE-Cy7 (clone 17A2), monoclonal, 1:100	BioLegend, USA	cat # 100220
rat anti-CD19-PE-Cy7 (clone 6D5), monoclonal, 1:100	BioLegend, USA	cat # 115519
rat anti-CD115-APC	Invitrogen	cat # 17-1152-82
goat anti-MerTK (biotinylated), 1:50	R&D Systems, Inc., USA	cat # BAF591
Streptavidin-PE, 1:200	BD Pharmingen, BD Biosciences, Heidelberg, Germany	cat # 554061

Supplemental Table 2
